# Assessing baseline dependency of anchor-based minimal important change (MIC): don’t stratify on the baseline score!

**DOI:** 10.1007/s11136-021-02886-2

**Published:** 2021-05-26

**Authors:** Berend Terluin, Ewa M. Roos, Caroline B. Terwee, Jonas B. Thorlund, Lina H. Ingelsrud

**Affiliations:** 1 Department of General Practice, Amsterdam Public Health Research Institute, Amsterdam UMC, Amsterdam, The Netherlands; 2 Department of Sports Science and Clinical Biomechanics, University of Southern Denmark, Odense, Denmark; 3 Department of Epidemiology and Data Science, Amsterdam Public Health Research Institute, Amsterdam UMC, Amsterdam, The Netherlands; 4 Department of Orthopaedic Surgery, Copenhagen University Hospital Hvidovre, Copenhagen, Denmark; 5 Research Unit for General Practice, Department of Public Health, University of Southern Denmark, Odense, Denmark

**Keywords:** Minimal important difference, Minimal important change, Baseline dependency, Mean change method, ROC method, Predictive modeling method

## Abstract

**Purpose:**

The minimal important change (MIC) of a patient-reported outcome measure (PROM) is often suspected to be baseline dependent, typically in the sense that patients who are in a poorer baseline health condition need greater improvement to qualify as minimally important. Testing MIC baseline dependency is commonly performed by creating two or more subgroups, stratified on the baseline PROM score. This study’s purpose was to show that this practice produces biased subgroup MIC estimates resulting in spurious MIC baseline dependency, and to develop alternative methods to evaluate MIC baseline dependency.

**Methods:**

Datasets with PROM baseline and follow-up scores and transition ratings were simulated with and without MIC baseline dependency. Mean change MICs, ROC-based MICs, predictive MICs, and adjusted MICs were estimated before and after stratification on the baseline score. Three alternative methods were developed and evaluated. The methods were applied in a real data example for illustration.

**Results:**

Baseline stratification resulted in biased subgroup MIC estimates and the false impression of MIC baseline dependency, due to redistribution of measurement error. Two of the alternative methods require a second baseline measurement with the same PROM or another correlated PROM. The third method involves the construction of two parallel tests based on splitting the PROM’s item set. Two methods could be applied to the real data.

**Conclusion:**

MIC baseline dependency should not be tested in subgroups based on stratification on the baseline PROM score. Instead, one or more of the suggested alternative methods should be used.

**Supplementary Information:**

The online version contains supplementary material available at 10.1007/s11136-021-02886-2.

## Introduction

The minimal important change (MIC) is defined as the smallest change in a patient-reported outcome measure (PROM) that is important to patients [1, 2]. Anchor-based MICs correspond to an external criterion (the “anchor”) of what constitutes a minimal important change for patients. This external criterion is often a transition question, asking patients to rate their perceived change between two moments in time [3]. Commonly used response options are “much better,” “a little better,” “unchanged,” “a little worse,” and “much worse.” The change of interest can be in the direction of improvement or deterioration. For simplicity, we will limit the present treatise to improvement, knowing that the case for deterioration is exactly the reverse. Three commonly applied methods to estimate anchor-based MICs are the mean change method, the receiver operating characteristic (ROC)-based method, and the predictive modeling method. According to the mean change method, the MIC is defined as the mean PROM change in the subgroup considered to have experienced a minimal important improvement, i.e., patients who have rated their condition as “a little better” [4]. According to the ROC-based method, the MIC is defined as the PROM change score threshold that optimally distinguishes improved from not-improved patients [5]. The predictive modeling method defines the MIC as the PROM change score that is equally likely to occur in the improved and not-improved groups [6, 7].

There seems to be a broad consensus in the literature that MICs often depend on the baseline PROM score, usually in the sense that patients who are in a relatively poor condition at baseline have greater MICs [8–11]. A plausible explanation is that patients who are in a relatively poor health state, need greater improvement to consider their change important, than patients who are in a better health condition [12]. The standard test for baseline dependency of the MIC is to estimate the MIC in severity subgroups based on baseline stratification, either split by the mean, median, or quartiles of the baseline score. However, baseline stratification results in subgroups with skewed baseline distributions. We suspected that this might result in biased subgroup MIC estimates.

We conducted three studies. In the first simulation study, we examined how standard baseline stratification affects the MIC estimation in the severity subgroups. In the second simulation study, we examined the performance of three alternative methods. In the third study, we compared the standard and alternative methods using a real dataset.

## Study 1

### Methods

**Fig. 1:**
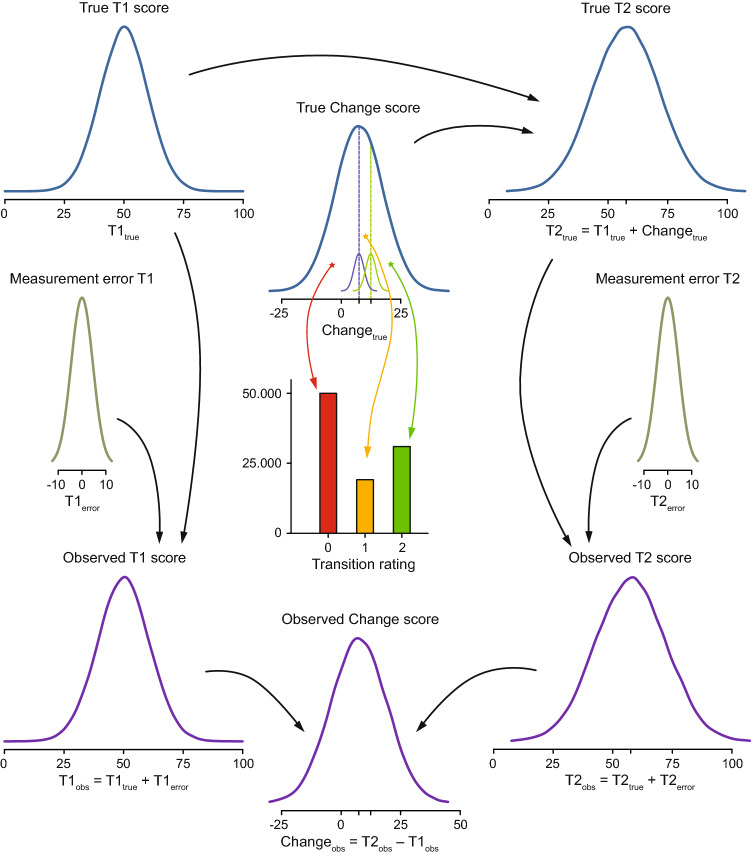
Graphical display of the simulation design. Simulated variables (depicted as density plots) were the true baseline (T1) score, the true change score, measurement error, and two transition rating thresholds (one between “no change” and “a little better,” and the other between “a little better” and “much better”). Mean thresholds are depicted by dashed lines; individual variability is indicated by small density plots overlying these lines. Transition ratings were derived by comparing the true change to the thresholds: rating “0” is “not-improved” (i.e., “unchanged,” “a little worse,” or “much worse”), rating “1” is “a little better,” rating “2” is “much better.” The following variables were derived from the simulated variables: the true follow-up (T2) score (the true T1 score plus the true change score), the observed T1 and T2 scores (the true T1 score plus measurement error of the T1 score, and the true T2 score plus measurement error of the T2 score, respectively), and the observed change score (the observed T2 score minus the observed T1 score)

We explored the effect of baseline stratification on the assessment of baseline dependency by simulating a dataset in which the MIC was not baseline dependent. The simulation started with the creation of normally distributed “true” (i.e., measurement error free) baseline (T1) scores with an arbitrary mean of 50 and an arbitrary standard deviation (SD) of 10, representing the latent baseline state expressed in the metric of the (simulated) PROM (Fig. 1). Next, normally distributed true change scores were created with an arbitrary mean of 7.5 and an SD of 10. The true change scores were given zero correlation with the true T1 scores. True follow-up scores (T2) were derived by adding the true change scores to the true T1 scores. The next step was to add measurement error (i.e., a random variable with a mean of zero) to the true T1 and T2 scores, and rounding the “observed” scores to integers. Equal error variance for T1 and T2 were created resulting in a reliability (i.e., the variance of the true score divided by the variance of the observed score) of the T1 scores of 0.85. The observed change scores were obtained by subtracting the observed T1 scores from the observed T2 scores. The final error scores of the T1, T2 and change scores were obtained by subtracting the true scores from the observed scores.

An important assumption underlying the use or transition ratings is that patients have their individual benchmarks by which they judge the magnitude and importance of a perceived change in their condition. More specifically, when asked to rate their change, e.g., as “much better,” “a little better,” “unchanged,” “a little worse,” or “much worse,” we assumed that patients compare their perceived change with a set of individual thresholds of what they consider to be a change that qualifies for “much better,” …, “much worse.” However, in the context of estimating anchor-based MIC values, only two specific thresholds were of interest. The first threshold, the one between “unchanged” and “a little better,” was used to dichotomize the group in improved and not-improved patients in order to estimate ROC-based and (adjusted) predictive modeling MICs [7]. The second threshold, the one between “a little better” and “much better,” was used to identify the group who had qualified their change as “a little better,” necessary to estimate the mean change MIC. To account for individual variability of the thresholds, we simulated two normal distributions of thresholds, the first with a mean of 7.5 and an SD of 1.5, and the second with a mean of 12.5 and an SD of 1.5. We let the thresholds be correlated (*r* = 0.75) to ensure that the second threshold was always greater than the first. Zero correlation was simulated between the true T1 scores and the thresholds to ensure that there was no baseline dependency of the MICs. The mean of the first threshold was chosen to equal the mean of the true change score in order to ensure that the proportion improved (i.e., patients rating “a little better” or “much better”) was 0.5. Proportions improved smaller or larger than 0.5 are known to cause bias in the ROC-based and predictive modeling MIC estimates [7], and we wished to avoid this complication. We simulated a sample of 100,000 patients to limit the influence of chance on the simulations.

The mean change MIC (MIC_mean_) was estimated as the mean observed change score in the “a little better” subgroup [4]. The ROC-based MIC (MIC_ROC_) was estimated by performing ROC analysis using the dichotomized transition ratings (improved versus not-improved) as the state variable and the observed change scores as the test variable [5]. The MIC_ROC_ was determined by the cutoff with the highest Youden index (i.e., the change score with the maximum sum of sensitivity and specificity). The predictive modeling MIC (MIC_predicted_) and the adjusted predictive modeling MIC (MIC_adjusted_) were calculated using the methods and formulas shown in Box 1 [6, 7].

**Box 1:** Formulas for calculating the predictive modeling MIC (predicted MIC) and the adjusted predictive modeling MIC (adjusted MIC)

MIC_Predicted_ = (logodds(imp) − *C*)/*B*	
MIC_Adjusted_ = MIC_Predicted_ − (0.090 + 0.103 × *Cor*) × SD_change_ × logodds(imp)	
Explanation:	
MIC_Predicted_ = Predictive modeling minimal important change	
logodds(imp) = logodds of improvement = natural logarithm of (proportion improved/(1 − proportion improved))	
*C* = Intercept coefficient of a logistic regression model with the PROM change score as the independent variable and the dichotomous transition rating (improved versus not-improved) as the dependent variable	
*B* = Regression coefficient of the logistic regression model as above	
MIC_Adjusted_ = Adjusted predictive modeling minimal important change	
*Cor* = Point-biserial correlation between the PROM change score and the dichotomous transition rating	
SD_change_ = standard deviation of the PROM change score	

We used two methods to split the group into a low- and a high-severity subgroup. The first method used the standard method based on median-splitting by the observed baseline scores. However, because median-splitting a normal distribution results in two highly non-normal distributions, we applied a second method using resampling to create two subgroups with normally distributed observed baseline scores. Resampling implied the pre-specification of two sets of normally distributed observed T1 scores with means of 41 and 59, respectively. These distributions were then “filled” with cases sampled from the original group. For instance, cases with a T1 score of 30 in the pre-specified low baseline distribution were filled by sampling cases (with replacement) from the original cases with the same T1 score, and this was done for all T1 score categories in both pre-specified T1 scores distributions. To our knowledge, resampling has never been used as an alternative for median-splitting on the baseline score. We applied it to enable stratification on the baseline score without creating heavily skewed subgroup samples.

### Results

In the total group, the MIC types based on the threshold between improved and not-improved (i.e., MIC_ROC_, MIC_predicted_, and MIC_adjusted_) were 7.5, which equaled both the mean observed change score and the mean of the first thresholds (Table 1, column 1) [7]. Moreover, MIC_ROC_ and MIC_predicted_ were not biased by the proportion improved because we simulated it to be 0.5 [7]. The MIC_mean_ value was 9.9, substantially higher than the other MIC values.

**Table 1:** Results before and after baseline stratification through median-splitting and resampling on the baseline score

Statistic	Total group	Median-split subgroups		Resampled subgroups	
		Low baseline	High baseline	Low baseline	High baseline
Proportion improved	0.50	0.50	0.50	0.50	0.50
Proportion “a little better”	0.19	0.19	0.19	0.19	0.19
Mean threshold 1	7.5	7.5	7.5	7.5	7.5
Mean threshold 2	12.5	12.5	12.5	12.5	12.5
Correlation T1-change score	0.00	0.00	− 0.00	− 0.00	− 0.00
Correlation T1-threshold 1	0.00	− 0.00	0.00	− 0.01	0.01
Mean observed T1 score	50.0	41.0	58.3	41.0	59.0
Mean error T1 score	− 0.0	− 1.4	1.2	− 1.3	1.4
Mean observed T2 score	57.5	49.9	64.6	49.9	65.2
Mean error T2 score	− 0.0	0.0	− 0.0	0.0	0.0
Mean observed change score	7.5	8.9	6.3	8.8	6.2
Mean error change score	0.0	1.4	− 1.3	1.3	− 1.4
MIC_mean_	9.9	11.3	8.6	11.3	8.6
MIC_ROC_	7.5	8.5	6.5	8.5	6.5
MIC_predicted_	7.5	8.9	6.3	8.9	6.2
MIC_adjusted_	7.5	8.9	6.3	8.9	6.2

**Fig. 2:**
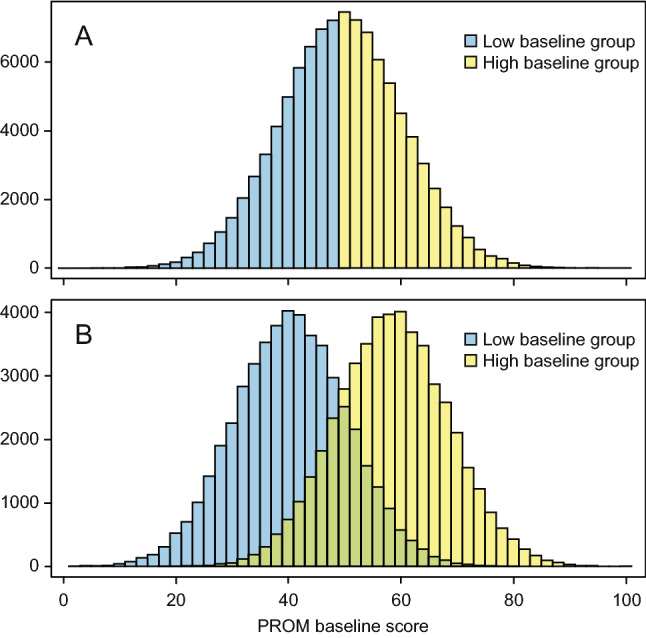
**A** Median-split subgroups; **B** resampled subgroups

As expected, median-splitting into two subgroups resulted in clearly different mean observed T1 scores (Table 1, columns 2–3) and highly skewed observed T1 scores (Fig. 2A). Because the thresholds were not correlated with the T1 score, the mean thresholds remained the same in both groups. Furthermore, because the change score was not correlated with the baseline score, the proportions improved and “a little better” also remained the same. Nevertheless, the MIC estimates were considerably greater in the low baseline subgroup than in the high baseline subgroup, equaling the mean observed change scores, but no longer the mean of the first thresholds. The resampling method resulted in subgroups with normally distributed baseline scores (Fig. 2B). Nevertheless, the method showed a picture very similar to the median-split method, including the false impression that the MICs were baseline dependent (Table 1, columns 4–5).

### Discussion

The simulations show that stratifying on the baseline score, either by median-splitting or by resampling, results in spurious baseline dependency of the MICs. Because resampling produced the same results as median-splitting, creating non-normal baseline scores can be ruled out as causing this phenomenon. We rather need to look at the mean change score. At this point it is important to recognize that the ROC and (adjusted) predictive modeling methods target a different MIC concept than the mean change method. The former methods target a MIC that represents the threshold change score in between not-improved and improved patients (i.e., the first threshold in our simulations), whereas the latter method targets a MIC that represents the mean change score in the group experiencing a minimal important improvement (i.e., a value roughly in between both thresholds in our simulations). If the mean change score equals the mean threshold between not-improved and improved, logically, in half of the cases the (true) change score will exceed this threshold, and consequently, the proportion improved will be 0.5 [7]. This is the case in the total group (Table 1). In the baseline stratified subgroups, this pattern is broken because the mean error component of the change scores is no longer zero, which spuriously increases (in the low baseline groups) or decreases (in the high baseline groups) the mean observed change score (Table 1). Because observed T1 scores consist of true T1 scores and error T1 scores, selecting a subgroup with relatively low (or high) observed T1 scores, selects a group not only with relatively low (or high) true T1 scores, but also with relatively low (or high) error T1 scores. This leads to a non-random redistribution of baseline measurement error across the subgroups. As a result, the mean baseline measurement error is non-zero in the subgroups (the mean baseline error is negative in the low baseline subgroup and positive in the high baseline subgroup, see Table 1). Because the observed change scores equal the observed T2 scores minus the observed T1 scores, the reduced (or elevated) mean error in the observed T1 scores is “transferred” to the observed change scores. This leads to an elevated mean error component of the change scores in the low baseline subgroup, and a reduced mean error component of the change scores in the high baseline subgroup. Consequently, the MICs reflect the spuriously elevated (or reduced) observed mean change scores, but not the mean thresholds (Table 1). This explains the false impression of baseline dependency in the ROC-based and (adjusted) predictive modeling MICs. The mean change MIC was 9.9 in the total group (i.e., a value roughly halfway in between both mean thresholds, 7.5 and 12.5). The mean change MIC, being the mean change score in a subgroup of patients, also reflected the spuriously elevated (or reduced) observed change scores in the subgroups based on baseline stratification.

## Study 2

### Methods

In Study 1 we found that spurious baseline dependency of the MIC results from stratifying on the baseline scores, which are subsequently used to calculate change scores. The obvious solution, therefore, seems to stratify on an independent assessment of the baseline state, which is not used to calculate change scores. In the following, we present three methods.

#### Method 1

An independent second baseline measurement with the same PROM would be ideal. Under the assumption that the second true baseline score would be the same as the initial true baseline score and that the error in the second measurement would not be correlated with the error in the initial measurement, stratifying on a second baseline measurement would select patients on the (initial) true baseline score only, and not on the (initial) error baseline score.

#### Method 2

Another possibility is using a baseline assessment with a different PROM that is correlated with the PROM of interest. If, for instance, the PROM of interest measures physical function, another PROM measuring activities of daily living might be used. One could also consider using a single-item global rating of severity as a different PROM. The correlation between the initial PROM and the other PROM should be large enough to obtain clearly different mean baseline scores on the initial PROM across the low and high baseline subgroups.

#### Method 3

A third possibility involves splitting the PROM’s item set and constructing two “parallel” test versions of the PROM based on different item sets. The parallel tests measure the same construct, while they do not share their measurement error. Method 3 actually represents a “trick” to create an independent second baseline measurement when only a single measurement with the PROM of interest is available. After stratifying the group on parallel test A, the MIC can be calculated for parallel test B, and vice versa. Importantly, the MICs, thus calculated, relate to the reduced scales of the parallel tests. MIC-A relates to the scale of parallel test A, and MIC-B to the scale of parallel test B. Given that the sum of the parallel tests represents the original PROM, the sum of MIC-A and MIC-B represent an estimate of the MIC related to the scale of the original PROM. Because there may be some variation depending on the exact division of the items across the parallel tests (and the internal consistency and unidimensionality of the PROM), we recommend repeating the random splitting of the item set a number of times (e.g., 5–10) and then take the average of the MIC estimates. Note that Method 3 is not available when the PROM consists of a single item (e.g., a visual analog scale).

Because methods 1 and 2 build on the same reasoning, using another PROM assessment that is correlated with the initial PROM assessment, we illustrate method 2 in the following. We added to the previous simulated example another PROM baseline measurement correlating 0.70 with the initial PROM baseline score. The other PROM measurement was used to median-split the group, after which the MICs were estimated in the low and high baseline subgroups. To examine whether this approach is capable of detecting baseline dependency if the MIC is truly baseline dependent, we used a different simulation and added baseline dependency of the MIC to the simulated example by adding a negative correlation between the first thresholds and the true T1 scores. A negative correlation implies that the thresholds between improved and not-improved are greater with smaller baseline scores. Thus, patients who are worse off (i.e., with lower baseline scores) need greater improvement to qualify it as “a little better” than patients who start at a better position. The true T1 scores and the first thresholds were set to correlate − 0.80. We added a small negative correlation (− 0.13) between the true T1 scores and the true change scores to ensure that the proportions improved remained about 0.50 in the subgroups.

To illustrate method 3, we simulated two sets of parallel test scores (A and B) representing the scores of two item sets, together making up the original PROM. So, the baseline A-scores plus the baseline B-scores resulted in the original PROM baseline score (with the same mean, SD, and reliability). Similarly, the change A-scores and the change B-scores added up to the original PROM change score, and the follow-up A-scores and the follow-up B-scores added up to the original PROM follow-up score. After median-splitting the group on the baseline B-scores, MICs were estimated for parallel test A in the low and high baseline subgroups, and vice versa. So, in each low or high baseline situation, two parallel test MICs were estimated, one based on parallel test A and the other based on parallel test B. Given that the sum of the parallel tests represented the original PROM, the sum of MIC-A and MIC-B represented an estimate of the MIC related to the original PROM scale. The simulations were performed under two conditions, one in which the MICs were independent of the baseline score, and the other in which the MICs were baseline dependent. Baseline dependency was simulated as in the simulation of method 2.

### Results

With method 2, in the absence of MIC baseline dependency, median-splitting the group on the baseline measurement of the other PROM resulted in two subgroups with different mean baseline scores, 43.9 versus 56.1 (Table 2, columns 2–3). Importantly, the mean error components of the baseline and change scores were now all close to zero in the low and high baseline subgroups. Consequently, the MICs reflected not only the mean change scores, but also the mean first thresholds. There was no false impression of MIC baseline dependency.

**Table 2:** Results before and after baseline stratification through median-splitting on an independent baseline measurement

Statistic	Without baseline-dependent MIC			With baseline-dependent MIC		
	Total	Low baseline	High baseline	Total	Low baseline	High baseline
Proportion improved	0.50	0.50	0.50	0.50	0.50	0.50
Proportion “a little better”	0.19	0.19	0.19	0.19	0.18	0.20
Mean threshold 1	7.5	7.5	7.5	7.5	8.2	6.8
Mean threshold 2	12.5	12.5	12.5	12.5	13.1	12.0
Correlation T1-change score	0.00	− 0.00	− 0.00	− 0.13	− 0.10	− 0.11
Correlation T1-threshold 1	0.00	− 0.00	0.00	− 0.80	− 0.73	− 0.72
Mean observed T1 score	50.0	43.9	56.1	50.0	43.9	56.1
Mean error T1 score	− 0.0	0.0	− 0.0	− 0.0	0.0	− 0.0
Mean observed T2 score	57.5	51.3	63.6	57.5	52.1	62.8
Mean error T2 score	− 0.0	− 0.0	− 0.0	− 0.0	− 0.0	− 0.0
Mean observed change score	7.5	7.5	7.6	7.5	8.2	6.8
Mean error change score	0.0	− 0.0	0.0	0.0	− 0.0	0.0
MIC_mean_	9.9	9.9	10.0	10.0	10.6	9.4
MIC_ROC_	7.5	7.5	7.5	7.5	8.5	7.5
MIC_predicted_	7.5	7.5	7.6	7.5	8.2	6.8
MIC_adjusted_	7.5	7.5	7.6	7.5	8.2	6.8

In the presence of baseline dependency of the MIC, stratifying on the other baseline measure, the low and high baseline groups not only differed in mean T1 scores but also in mean thresholds, in accordance with the simulated baseline dependency of the MIC (Table 2, columns 5–6). In both low and high baseline subgroups, MIC_predicted_ and MIC_adjusted_ equaled both the mean observed change score and the mean of the first thresholds. MIC_ROC_ followed this pattern but showed some deviance from the mean observed change score and the mean of the first thresholds, first, because MIC_ROC_ can only take on values midway between discrete change scores (e.g., 6.5, 7.5, 8.5), and second, because MIC_ROC_ proves to be less precise than MIC_predicted_ and MIC_adjusted_ [6]. MIC_mean_ also confirmed the existence of baseline dependency.

For method 3, the results for the total scale (Table 3, columns 1 and 6) demonstrate that the parallel tests indeed added up to the original PROM scale. In the absence of MIC baseline dependency, the MIC estimates did not differ across low and high baseline groups (Table 3, columns 2–3, and 4–5). In contrast, in the presence of MIC baseline dependency, the MICs were found consistently higher in the low baseline groups than in the high baseline groups (Table 3, columns 7–8, and 9–10). The adjusted MIC-A for the low baseline group was 4.2, which was the same for MIC-B in the low baseline group. These MICs added up to 8.4, which was identical to the sum of the mean observed change scores and the mean of the first thresholds in the low baseline subgroups. Similarly, the high baseline MICs (3.4 and 3.4) added up to 6.8, which was identical (within rounding error) to the sum of the mean observed change scores and the mean of the first thresholds in the high baseline subgroups.

**Table 3:** Results before and after baseline stratification through median-splitting on one of the parallel tests’ baseline score (item-split method)

Statistic	Without baseline-dependent MIC					With baseline-dependent MIC				
	Total	Parallel test A^a^		Parallel test B^b^		Total	Parallel test A^a^		Parallel test B^b^	
		Low BL^c^	High BL^c^	Low BL^c^	High BL^c^		Low BL^c^	High BL^c^	Low BL^c^	High BL^c^
Proportion improved	0.50	0.50	0.50	0.50	0.50	0.50	0.50	0.50	0.50	0.50
Proportion “a little better”	0.19	0.19	0.19	0.19	0.19	0.19	0.18	0.20	0.18	0.20
Mean threshold 1^d^	7.5	7.5	7.5	7.5	7.5	7.5	8.4	6.7	8.4	6.7
Mean threshold 2^d^	12.5	12.5	12.5	12.5	12.5	12.5	13.2	11.9	13.2	11.9
Correlation T1-change score	0.00	0.00	0.00	− 0.00	− 0.00	− 0.13	− 0.11	− 0.09	− 0.09	− 0.10
Correlation T1-threshold 1	0.00	− 0.00	0.00	− 0.00	0.00	− 0.80	− 0.69	− 0.70	− 0.69	− 0.70
Mean observed T1 score	50.0	21.3	28.2	21.3	28.2	50.0	21.3	28.2	21.3	28.2
Mean error T1 score	− 0.0	0.0	0.0	0.0	− 0.0	− 0.0	− 0.0	− 0.0	0.0	− 0.0
Mean observed T2 score	57.5	25.1	31.9	25.1	32.0	57.5	25.6	31.5	25.6	31.5
Mean error T2 score	0.0	0.0	0.0	0.0	− 0.0	− 0.0	− 0.0	− 0.0	− 0.0	0.0
Mean observed change score	7.5	3.8	3.7	3.7	3.8	7.5	4.2	3.4	4.2	3.4
Mean error change score	− 0.0	− 0.0	− 0.0	− 0.0	0.0	0.0	0.0	0.0	− 0.0	0.0
MIC_mean_	9.9	4.9	4.9	4.9	4.9	10.0	5.4	4.7	5.3	4.7
MIC_ROC_	7.5	3.5	3.5	3.5	3.5	7.5	4.5	3.5	4.5	3.5
MIC_predicted_	7.5	3.7	3.7	3.7	3.7	7.5	4.2	3.4	4.2	3.4
MIC_adjusted_	7.5	3.7	3.7	3.7	3.8	7.5	4.2	3.4	4.2	3.4

^a^Results for parallel test A, after stratification on the baseline measurement of parallel test B

^b^Results for parallel test B, after stratification on the baseline measurement of parallel test A

^c^Low BL: low baseline; High BL: high baseline

^d^Thresholds are in the metric of the total scale

### Discussion

The proposed methods to avoid spurious baseline dependency of the MIC rest on selecting severity subgroups on the basis of a baseline measure that is not involved in the calculation of the MIC or, put more specifically, a baseline measure whose measurement error does not end up in the change score that is used to calculate the MIC. The independent baseline measurement with the same PROM (method 1, i.e., a kind of “retest” measurement) might be ideal but will often not be feasible in practice. An independent baseline measurement with another PROM, which is correlated to the PROM of interest (method 2) can also be used to select the low and high baseline subgroups. The higher the correlation between the baseline PROM of interest and the other PROM, the better the groups will be separated on the baseline severity of the construct of interest. If the correlation is relatively low, the approach may fail to clearly separate the subgroups on baseline severity, and consequently may fail to detect a truly existing MIC baseline dependency (type II error). However, the approach protects against spuriously detecting MIC baseline dependency where it is truly absent (type I error). A significance test can be based on the calculation of bootstrap intervals (as illustrated in Study 3).

## Study 3

### Methods

To exemplify the MIC baseline dependency methods, we analyzed a dataset from a study of 614 patients undergoing knee arthroscopy (see the Supplementary material, section 1, for a more detailed study description) [13]. Patients completed the Knee injury and Osteoarthritis Outcome Score (KOOS) before and 3 months after surgery. The KOOS comprises five scales, measuring Pain, Symptoms, Activities of Daily Living (ADL), Sport and Recreation (Sport/Rec), and Quality Of Life (QOL) [14]. The items are scored 0 (no problems) to 4 (extreme problems), and summary scores are reversed and transformed to a 0–100 (extreme—no problems) scale (KOOS scoring guide, 2012, http://www.koos.nu/). At 3 months of follow-up, patients also answered transition questions, specifically relating to the separate KOOS scales. The response options constituted a 7-point scale from 1 (“Better, an important improvement”) to 7 (“Worse, an important deterioration”).

The mean change score of the subgroup scoring 2 (“Somewhat better, but enough to be an important improvement”) was taken as MIC_mean_. In order to calculate the other anchor-based MICs, the transition ratings were dichotomized into “importantly improved” (transition ratings 1 and 2) and “not (importantly) improved” (transition ratings 3–7).

Baseline dependency of the MICs was assessed using the standard method and the alternative methods 2 and 3. For Method 2, we used one of the other KOOS scales that correlated highest with the scale under study. For Method 3, the random splitting of the item set was performed five times and the results were averaged. We used bootstrapping (1000 bootstrap samples) to generate 95% confidence intervals (95% CI) around the MIC estimates.

### Results and discussion

The standard method yielded statistically significantly higher MIC values for the low baseline subgroups than for the high baseline subgroups, across all five scales and all four MIC types (Supplementary material, section 1, Tables S1–S5). Methods 2 and 3 suggested true MIC baseline dependency of the Pain, Symptoms, ADL, and Sport/Rec scales, but not of the QOL scale.

We recently published MIC values for the KOOS scales based on these data [15]. Had we tested for baseline dependency of these MICs using the standard baseline stratification method, we would erroneously have concluded that the MIC for the QOL scale was baseline dependent. Using the alternative methods, we have now established that the MIC of the QOL scale is not baseline dependent while the MICs of the other scales are. With respect to the precision of the MIC estimates, Method 3 appeared to perform as good as or even better than Method 2.

## General discussion

Over the past few decades, several authors have suggested that the MIC was baseline dependent based on stratification on the baseline PROM score. We have shown that this method is bound to produce spurious results because selecting subgroups based on high (or low) baseline PROM scores not only selects subgroups with high (or low) baseline true scores, but also on high (or low) baseline error scores, thus non-randomly redistributing the measurement error in the PROM change score across the subgroups. As a result, the mean change score in the low baseline group is spuriously increased and the mean change score in the high baseline group is spuriously decreased. When using commonly used MIC methods such as the mean change method, the ROC-based method, and the (adjusted) predictive modeling method, the spuriously increased or decreased change scores translate into spuriously increased MIC estimates in the low baseline group and spuriously decreased MIC estimates in the high baseline group. This yields a false impression of baseline dependency of the MIC even if there is no true MIC baseline dependency. With the three alternative methods we have proposed, this pitfall is avoided.

With respect to the validity of MIC estimates, we have to post a warning when using the proposed methods to assess baseline dependency. We simulated “ideal” data with normally distributed PROM (change) scores, and transition ratings that perfectly reflected change in the construct of interest. However, real data are often less ideal with skewed PROM (change) scores and transition ratings sometime poorly correlate with the change scores. Under these less than ideal circumstances, MIC estimates may become biased and/or imprecise (this is a largely understudied area). Assessing baseline dependency under such circumstances is likely to produce biased and/or imprecise MIC estimates as well. However, assessing baseline dependency using the “wrong” method will almost certainly add spurious baseline dependency to the already biased/imprecise results. Using the proper methods may result in correct conclusions regarding baseline dependency of otherwise still biased/imprecise MIC estimates.

MIC values likely vary across individuals for various reasons. One of the reasons might be baseline severity. Other potential reasons are gender, age, and treatment (e.g., surgical or non-surgical). Knowing some of the causes for MIC variability might provide more precise MIC estimates for specific situations.

## Conclusion

Stratification on the baseline PROM score and estimating MICs in the low and high baseline subgroups yields a false impression of MIC baseline dependency. We have described three alternative methods to create low and high baseline subgroups, which do not carry the risk of biased MIC estimation.

## Supplementary Information

Below is the link to the electronic supplementary material.

Supplementary file1 (DOCX 116 kb)

## Data Availability

The R-code used to simulate and analyze samples is provided in the Supplementary material. There are no patient data (as used in study 3) available.

## Code availability

The R-code for assessing baseline dependency of an MIC, using Method 3 (the item-split method), is provided in the Supplementary material.
