# Supplementary material for: Assessing baseline dependency of anchor-based minimal important change (MIC): don’t stratify on the baseline score!
Source: Qual Life Res. 2021 May 26;30(10):2773–82. doi: 10.1007/s11136-021-02886-2 (PMC8481187; doi:10.1007/s11136-021-02886-2)
Supplement: Supplementary file 1 — Supplementary file1 (DOCX 116 kb) [file 11136_2021_2886_MOESM1_ESM.docx]

**Supplementary material**

**Article:** Assessing baseline dependency of anchor-based minimal important change (MIC): don’t stratify on the baseline score!

**Journal:** Quality of Life Research

**Authors:** Berend Terluin*, Ewa M Roos, Caroline B Terwee, Jonas B Thorlund, Lina H Ingelsrud

* Corresponding author

Department of General Practice, Amsterdam Public Health research institute, Amsterdam UMC, Amsterdam, The Netherlands

Email address: b.terluin@amsterdamumc.nl

1. MIC baseline dependency of the KOOS scales (study 3) 2

2. R-code used for the simulations 9

2.1. R-code for Table 1 9

2.2. R-code for Table 2 20

2.3. R-code for Table 3 (columns 1-5) 33

2.4. R-code for Table 3 (columns 6-10) 43

3. R-code for the item-split method to assess MIC baseline dependency 53

Section 1

**MIC baseline dependency of the KOOS scales (study 3)**

*Introduction and Methods*

To exemplify the MIC baseline dependency methods, we analyzed a dataset from a study of 614 patients (mean ± SD age 50 ± 13, and 44% female) undergoing knee arthroscopy for a meniscal tear. Patients completed the Knee injury and Osteoarthritis Outcome Score (KOOS) before and 3 months after surgery. The KOOS comprises 5 scales, measuring Pain (9 items, Cronbach’s alpha 0.89), Symptoms (7 items, alpha 0.70), Activities of Daily Living (ADL, 17 items, alpha 0.96), Sport and Recreation (Sport/Rec, 5 items, alpha 0.87) and Quality Of Life (QOL, 4 items, alpha 0.51). KOOS items are scored 0 (no problems) to 4 (extreme problems), and summary scores are reversed and transformed to a 0-100 (extreme – no problems) scale (KOOS scoring guide, 2012, http://www.koos.nu/). At 3 months follow-up patients also answered transition questions, specifically relating to the separate KOOS scales. The five transition questions asked about the degree and importance of change in subscale domain-specific health-state from before to after surgery. The response options formed a 7-point scale:

1: Better, an important improvement,

2: Somewhat better, but enough to be an important improvement,

3: Very small change, not enough to be an important improvement,

4: About the same,

5: Very small change, not enough to be an important deterioration,

6: Somewhat worse, but enough to be an important deterioration,

7: Worse, an important deterioration.

The mean change score of the subgroup scoring 2 (“Somewhat better, but enough to be an important improvement”) was taken as MIC_mean_. In order to calculate the other anchor-based MICs, the transition ratings were dichotomized into “importantly improved” (transition ratings 1 and 2) and “not (importantly) improved (transition ratings 3-7). MIC_ROC_, MIC_predicted_ and MIC_adjusted_ were calculated as described in the paper.

Baseline dependency of the MICs was estimated using the standard method (denoted “Method 0”, see Study 1 in the paper) and two of the alternative methods outlined under Study 2 in the paper. Because no second baseline measurements with the same KOOS scales were available, Method 1 could not be applied. For Method 2, we used one of the other KOOS scales, the one with the highest correlation with the scale under study, to median-split the group in severity subgroups. For Method 3, we used the procedure outlined under Study 2 in the paper and the R-code provided in section 3 of this Supplementary material. The analysis was performed on the raw (i.e., untransformed) (item) scores, and the results were afterwards transformed to the 0-100 scales. Splitting the item set was performed randomly five times and the results were averaged. However, as the QOL scale only consists of 4 items, only three different item-split sets were possible, of which the results were averaged. We used bootstrap sampling (n=1000) to generate 95% confidence intervals (95% CI) around the MIC estimates and the MIC differences across low and high baseline subgroups.

*Results*

The Spearman correlations between the KOOS change scores and the corresponding 7-point transition ratings were 0.58 (Pain), 0.55 (Symptoms), 0.53 (ADL), 0.59 (Sport/Rec), and 0.60 (QOL).

For all KOOS scales, using Method 0 (the “wrong” baseline stratification method) yielded statistically significantly higher MIC values for the low baseline subgroups than for the high baseline subgroups, across all five scales and all four MIC types (Tables S1 - S5). Regarding Method 2, the ADL scale had the highest correlations with the Pain, Sport/Rec and QOL scales (correlations 0.86, 0.67, and 0.58 respectively) and was used as the second measurement to stratify the group to assess MIC baseline dependency of the Pain, Sport/Rec and QOL scales. The Pain scale had the highest correlations with the Symptoms and ADL scales (correlations 0.66, and 0.86 respectively) and was similarly used to assess MIC baseline dependency of the Symptoms and ADL scales.

When applying the two alternative methods (Methods 2 and 3), we found that all MIC values were significantly different across the low and high baseline subgroups for the Pain and ADL scales (Tables S1 and S3). In case of the Symptoms and Sport/Rec scales (Tables S2 and S4) MIC_predicted_ and MIC_adjusted_ were significantly different across the severity subgroups (and MIC_mean_ also in three out of four estimations), whereas MIC_ROC_ was not significantly different. For the QOL scale, MIC estimates did not significantly differ between the low and high baseline subgroups (Table S5).

*Discussion*

The standard baseline stratification method (Method 0) suggested that the MIC for the QOL scale was baseline dependent. Using the alternative methods, we have now established that the MIC of the QOL scale is not baseline dependent, while the MICs of the other KOOS scales are. The discrepancy between the ROC-based MIC and the (adjusted) predictive MICs must be attributed to the relatively poor precision of the ROC-based MIC, expressing itself by wider confidence intervals than the other MIC estimates.

It is remarkable how well Method 3 (the item-split method) performed in comparison with Method 2, with respect to the precision of the MIC estimates. Considering the smaller confidence intervals of the low-high MIC differences, Method 3 appeared somewhat more precise than Method 2 in case of the Pain, Symptoms, ADL, and Sport/Rec scales (Tables S1-S4). In case of the QOL scale, a scale with only 4 items, Methods 3 and Method 2 appeared to produce similar precision (Table S5). Apparently, Method 3 does not yield less reliable results than Method 2. Maybe Method 3 performs comparably to Method 1, but we were not able to test this because a second baseline measurement was not available.

**Table S1. Assessment of baseline dependency of four types of MICs for the KOOS Pain scale, MIC estimates and 95% CIs by statistical method**

| **Method** | **Low baseline subgroup** | | | **High baseline subgroup** | | | **Low-high difference** | |
| --- | --- | --- | --- | --- | --- | --- | --- | --- |
|  | MIC | 95% CI | Proportion improved | MIC | 95% CI | Proportion improved | ΔMIC | 95% CI |
| **Method 0** ^a^ |  |  |  |  |  |  |  |  |
| MIC_mean_ | 27.4 | 24.5; 30.6 | 0.56 | 9.2 | 6.6; 12.5 | 0.65 | 18.3 | 14.0; 22.1 |
| MIC_ROC_ | 22.2 | 13.9; 23.6 | 0.56 | 8.3 | 2.8; 13.9 | 0.65 | 13.9 | 4.2; 20.8 |
| MIC_predicted_ | 20.8 | 19.1; 23.0 | 0.56 | 6.4 | 5.0; 8.3 | 0.65 | 14.4 | 11.9; 16.7 |
| MIC_adjusted_ | 20.1 | 18.2; 22.3 | 0.56 | 5.0 | 3.4; 7.0 | 0.65 | 15.1 | 12.4; 17.6 |
| **Method 2** ^b^ |  |  |  |  |  |  |  |  |
| MIC_mean_ | 26.4 | 22.2; 29.5 | 0.55 | 11.2 | 8.3; 13.9 | 0.65 | 15.2 | 10.2; 19.5 |
| MIC_ROC_ | 19.4 | 12.5; 23.6 | 0.55 | 8.3 | 2.8; 11.1 | 0.65 | 11.1 | 4.2; 18.1 |
| MIC_predicted_ | 19.1 | 16.7; 20.9 | 0.55 | 8.2 | 6.5; 10.0 | 0.65 | 10.9 | 8.0; 13.3 |
| MIC_adjusted_ | 18.4 | 15.9; 20.3 | 0.55 | 6.7 | 4.8; 8.5 | 0.65 | 11.7 | 8.4; 14.1 |
| **Method 3** ^c^ |  |  |  |  |  |  |  |  |
| MIC_mean_ | 24.8 | 23.7; 26.5 | 0.57 | 12.5 | 11.4; 13.7 | 0.63 | 12.4 | 10.4; 14.5 |
| MIC_ROC_ | 20.6 | 16.7; 25.0 | 0.57 | 8.3 | 2.8; 13.9 | 0.63 | 12.2 | 2.8; 16.7 |
| MIC_predicted_ | 19.4 | 18.8; 20.8 | 0.57 | 8.4 | 7.6; 8.9 | 0.63 | 11.1 | 10.1; 12.6 |
| MIC_adjusted_ | 18.6 | 17.9; 20.0 | 0.57 | 6.9 | 6.2; 7.4 | 0.63 | 11.7 | 10.6; 13.2 |

^a^ Method 0: Median split by the baseline score of the PRO scale under study

^b^ Method 2: Median split by the baseline score of another PRO scale

^c^ Method 3: Median split by the baseline score of one of the parallel tests (item-split method)

**Table S2. Assessment of baseline dependency of four types of MICs for the KOOS Symptoms scale, MIC estimates and 95% CIs by statistical method**

| **Method** | **Low baseline subgroup** | | | **High baseline subgroup** | | | **Low-high difference** | |
| --- | --- | --- | --- | --- | --- | --- | --- | --- |
|  | MIC | 95% CI | Proportion improved | MIC | 95% CI | Proportion improved | ΔMIC | 95% CI |
| **Method 0** ^a^ |  |  |  |  |  |  |  |  |
| MIC_mean_ | 19.5 | 15.4; 23.3 | 0.56 | 5.3 | 2.2; 8.1 | 0.59 | 14.2 | 9.7; 18.9 |
| MIC_ROC_ | 16.1 | 12.5; 21.4 | 0.56 | 3.6 | -1.8; 8.9 | 0.59 | 12.5 | 5.4; 21.4 |
| MIC_predicted_ | 16.0 | 14.0; 17.7 | 0.56 | 2.6 | 0.9; 4.2 | 0.59 | 13.4 | 11.0; 15.7 |
| MIC_adjusted_ | 15.3 | 13.2; 17.1 | 0.56 | 1.7 | -0.1; 3.4 | 0.59 | 13.5 | 11.0; 16.1 |
| **Method 2** ^b^ |  |  |  |  |  |  |  |  |
| MIC_mean_ | 16.2 | 12.5; 20.1 | 0.55 | 7.5 | 4.8; 11.2 | 0.60 | 8.6 | 3.7; 13.4 |
| MIC_ROC_ | 14.3 | 3.6; 25.0 | 0.55 | 7.1 | -3.6; 12.5 | 0.60 | 7.1 | -3.6; 21.4 |
| MIC_predicted_ | 12.3 | 10.3; 14.4 | 0.55 | 5.0 | 3.4; 6.8 | 0.60 | 7.2 | 4.5; 9.9 |
| MIC_adjusted_ | 11.7 | 9.6; 14.0 | 0.55 | 4.0 | 2.3; 6.0 | 0.60 | 7.7 | 4.9; 10.4 |
| **Method 3** ^c^ |  |  |  |  |  |  |  |  |
| MIC_mean_ | 15.5 | 13.5; 17.1 | 0.56 | 8.8 | 7.7; 10.2 | 0.58 | 6.7 | 3.3; 9.3 |
| MIC_ROC_ | 13.6 | 7.1; 17.9 | 0.56 | 7.1 | 0.0; 14.3 | 0.58 | 6.4 | -3.6; 14.3 |
| MIC_predicted_ | 12.1 | 10.4; 13.9 | 0.56 | 5.9 | 4.7; 6.8 | 0.58 | 6.3 | 3.6; 8.9 |
| MIC_adjusted_ | 11.4 | 9.6; 12.9 | 0.56 | 4.9 | 3.7; 5.9 | 0.58 | 6.5 | 3.7; 9.1 |

^a^ Method 0: Median split by the baseline score of the PRO scale under study

^b^ Method 2: Median split by the baseline score of another PRO scale

^c^ Method 3: Median split by the baseline score of one of the parallel tests (item-split method)

**Table S3. Assessment of baseline dependency of four types of MICs for the KOOS ADL scale, MIC estimates and 95% CIs by statistical method**

| **Method** | **Low baseline subgroup** | | | **High baseline subgroup** | | | **Low-high difference** | |
| --- | --- | --- | --- | --- | --- | --- | --- | --- |
|  | MIC | 95% CI | Proportion improved | MIC | 95% CI | Proportion improved | ΔMIC | 95% CI |
| **Method 0** ^a^ |  |  |  |  |  |  |  |  |
| MIC_mean_ | 24.5 | 21.2; 27.3 | 0.53 | 8.8 | 6.6; 11.3 | 0.57 | 15.6 | 11.6; 19.5 |
| MIC_ROC_ | 20.6 | 15.4; 25.7 | 0.53 | 8.1 | 0.7; 8.1 | 0.57 | 12.5 | 8.8; 19.9 |
| MIC_predicted_ | 20.7 | 18.6; 22.5 | 0.53 | 6.3 | 4.8; 7.5 | 0.57 | 14.4 | 12.3; 16.5 |
| MIC_adjusted_ | 20.4 | 18.2; 22.1 | 0.53 | 5.8 | 4.0; 7.1 | 0.57 | 14.6 | 12.3; 17.0 |
| **Method 2** ^b^ |  |  |  |  |  |  |  |  |
| MIC_mean_ | 24.0 | 20.7; 27.2 | 0.53 | 9.3 | 7.3; 12.4 | 0.57 | 14.7 | 10.0; 18.3 |
| MIC_ROC_ | 20.6 | 19.1; 25.0 | 0.53 | 7.4 | 0.7; 8.1 | 0.57 | 13.2 | 11.0; 22.1 |
| MIC_predicted_ | 19.3 | 17.5; 21.5 | 0.53 | 6.8 | 5.5; 8.6 | 0.57 | 12.5 | 10.0; 14.7 |
| MIC_adjusted_ | 18.9 | 17.1; 21.1 | 0.53 | 6.3 | 4.8; 8.2 | 0.57 | 12.7 | 10.0; 15.2 |
| **Method 3** ^c^ |  |  |  |  |  |  |  |  |
| MIC_mean_ | 24.2 | 23.3; 24.6 | 0.53 | 10.0 | 9.1; 10.6 | 0.57 | 14.1 | 12.9; 15.2 |
| MIC_ROC_ | 19.4 | 17.6; 23.5 | 0.53 | 7.1 | 2.9; 8.8 | 0.57 | 12.4 | 10.3; 19.1 |
| MIC_predicted_ | 20.2 | 19.4; 20.4 | 0.53 | 6.8 | 6.4; 7.2 | 0.57 | 13.4 | 12.5; 13.7 |
| MIC_adjusted_ | 19.9 | 19.1; 20.1 | 0.53 | 6.1 | 5.7; 6.6 | 0.57 | 13.8 | 12.8; 14.1 |

^a^ Method 0: Median split by the baseline score of the PRO scale under study

^b^ Method 2: Median split by the baseline score of another PRO scale

^c^ Method 3: Median split by the baseline score of one of the parallel tests (item-split method)

**Table S4. Assessment of baseline dependency of four types of MICs for the KOOS Sport/Rec scale, MIC estimates and 95% CIs by statistical method**

| **Method** | **Low baseline subgroup** | | | **High baseline subgroup** | | | **Low-high difference** | |
| --- | --- | --- | --- | --- | --- | --- | --- | --- |
|  | MIC | 95% CI | Proportion improved | MIC | 95% CI | Proportion improved | ΔMIC | 95% CI |
| **Method 0** ^a^ |  |  |  |  |  |  |  |  |
| MIC_mean_ | 37.4 | 32.1; 42.4 | 0.35 | 19.2 | 15.8; 23.6 | 0.51 | 18.2 | 11.2; 24.3 |
| MIC_ROC_ | 22.5 | 22.5; 30.0 | 0.35 | 15.0 | 10.0; 15.0 | 0.51 | 7.5 | 7.5; 17.5 |
| MIC_predicted_ | 23.2 | 20.4; 25.9 | 0.35 | 9.4 | 7.2; 12.1 | 0.51 | 13.8 | 9.9; 17.2 |
| MIC_adjusted_ | 25.4 | 22.5; 28.4 | 0.35 | 9.2 | 6.7; 12.1 | 0.51 | 16.1 | 12.1; 19.9 |
| **Method 2** ^b^ |  |  |  |  |  |  |  |  |
| MIC_mean_ | 31.1 | 24.9; 35.9 | 0.36 | 23.5 | 19.6; 27.9 | 0.50 | 7.6 | -0.4; 13.7 |
| MIC_ROC_ | 25.0 | 10.0; 27.5 | 0.36 | 15.0 | 12.5; 22.5 | 0.50 | 10.0 | -5.1; 15.0 |
| MIC_predicted_ | 18.7 | 15.8; 21.1 | 0.36 | 13.4 | 11.3; 16.1 | 0.50 | 5.3 | 1.1; 8.4 |
| MIC_adjusted_ | 20.8 | 17.5; 23.2 | 0.36 | 13.4 | 10.9; 16.1 | 0.50 | 7.4 | 2.9; 10.7 |
| **Method 3** ^c^ |  |  |  |  |  |  |  |  |
| MIC_mean_ | 35.7 | 33.7; 36.2 | 0.36 | 21.2 | 20.8; 22.4 | 0.50 | 14.5 | 11.3; 15.4 |
| MIC_ROC_ | 19.0 | 15.0; 30.0 | 0.36 | 14.0 | 10.0; 20.0 | 0.50 | 5.0 | -5.0; 20.0 |
| MIC_predicted_ | 22.1 | 21.6; 22.2 | 0.36 | 11.6 | 11.2; 12.3 | 0.50 | 10.5 | 9.3; 10.9 |
| MIC_adjusted_ | 24.3 | 23.8; 24.5 | 0.36 | 11.7 | 11.3; 12.4 | 0.50 | 12.6 | 11.5; 13.0 |

^a^ Method 0: Median split by the baseline score of the PRO scale under study

^b^ Method 2: Median split by the baseline score of another PRO scale

^c^ Method 3: Median split by the baseline score of one of the parallel tests (item-split method)

**Table S5. Assessment of baseline dependency of four types of MICs for the KOOS QOL scale, MIC estimates and 95% CIs by statistical method**

| **Method** | **Low baseline subgroup** | | | **High baseline subgroup** | | | **Low-high difference** | |
| --- | --- | --- | --- | --- | --- | --- | --- | --- |
|  | MIC | 95% CI | Proportion improved | MIC | 95% CI | Proportion improved | ΔMIC | 95% CI |
| **Method 0** ^a^ |  |  |  |  |  |  |  |  |
| MIC_mean_ | 19.1 | 15.7; 25.4 | 0.49 | 7.7 | 4.2; 11.4 | 0.51 | 11.4 | 6.6; 17.5 |
| MIC_ROC_ | 9.4 | 9.4; 21.9 | 0.49 | 9.4 | 3.1; 15.6 | 0.51 | 0.0 | 0.0; 12.5 |
| MIC_predicted_ | 13.4 | 11.5; 16.9 | 0.49 | 4.7 | 3.0; 6.5 | 0.51 | 8.7 | 6.3; 12.0 |
| MIC_adjusted_ | 13.4 | 11.5; 17.6 | 0.49 | 4.5 | 2.8; 6.4 | 0.51 | 8.9 | 6.3; 13.0 |
| **Method 2** ^b^ |  |  |  |  |  |  |  |  |
| MIC_mean_ | 12.7 | 9.5; 16.3 | 0.48 | 14.9 | 10.5; 18.5 | 0.52 | -2.2 | -6.7; 3.8 |
| MIC_ROC_ | 9.4 | 3.1; 15.6 | 0.48 | 9.4 | 3.1; 15.6 | 0.52 | 0.0 | -6.3; 6.3 |
| MIC_predicted_ | 9.1 | 7.0; 10.8 | 0.48 | 8.6 | 6.9; 10.5 | 0.52 | 0.5 | -2.5; 2.9 |
| MIC_adjusted_ | 9.3 | 7.2; 11.0 | 0.48 | 8.4 | 6.5; 10.4 | 0.52 | 0.9 | -2.0; 3.4 |
| **Method 3** ^c^ |  |  |  |  |  |  |  |  |
| MIC_mean_ | 13.2 | 9.2; 17.4 | 0.45 | 13.9 | 11.2; 16.9 | 0.53 | -0.7 | -5.4; 4.0 |
| MIC_ROC_ | 10.4 | 6.3; 18.8 | 0.45 | 14.6 | 8.3; 16.7 | 0.53 | -4.2 | -6.3; 8.3 |
| MIC_predicted_ | 10.2 | 8.0; 12.4 | 0.45 | 8.5 | 7.1; 9.9 | 0.53 | 1.7 | -0.8; 4.1 |
| MIC_adjusted_ | 10.8 | 8.4; 13.0 | 0.45 | 8.2 | 6.7; 9.7 | 0.53 | 2.6 | -0.1; 5.1 |

^a^ Method 0: Median split by the baseline score of the PRO scale under study

^b^ Method 2: Median split by the baseline score of another PRO scale

^c^ Method 3: Median split by the baseline score of one of the parallel tests (item-split method)

Section 2

**R-code used for the simulations**

############################################################################

### ###

### SECTION 2.1. BASELINE DEPENDENCY SIMULATIONS TABLE 1 ###

### ###

############################################################################

library(pROC)

library(ggplot2)

library(psych)

rm(list=ls(all=TRUE))

set.seed (12345)

## --------------------------------

## Set sample parameters

sample.size.set <- 100000

## parameter controlling the sample size

mn.xt1.set <- 50

## mean true PROM score at T1

sd.xt1.set <- 0.20

## parameter controlling the SD of the true PROM score at T1;

## values are ratios of the SD of the true PROM score to the mean

## of the true PROM score at T1

rr.xt.set <- 0.85

## reliability coefficient of PROM score (T1)

mn.imic.set <- 0.75

## parameter controlling the mean iMIC (= genuine MIC; gMIC);

## values are ratios of the mean iMIC to the SD of the PROM score at T1,

## reflecting the relative magnitude of the effect needed to qualify as

## genuine MIC (gMIC): 0.5 moderate, 0.8 large effect

sd.imic.set <- 0.2

## parameter controlling the SD of the iMICs

## values are proportions of the mean iMIC (or gMIC)

## TAKE CARE: the some iMIC values should not approach zero as this

## will not be realistic

sd.xtc.set <- 1

## parameter controlling the SD of the true PROM change score (T2-T1),

## i.e. variability of change;

## values are ratios of the SD of the true change score to the SD of the

## true PROM score at T1

mn.xtc.set <- 0

## parameter controlling the mean true PROM change score;

## values express the difference between the mean true change score and

## the gMIC in SD units of the true change score;

## note: the position of the mean true change score relative to the gMIC

## determines the PROPORTION IMPROVED

cor.t1.xtc.set <- 0

## parameter controlling the correlation between true T1 scores and the

## true change scores; values represent correlation coefficients

cor.t1.imic.set <- 0

## parameter controlling the correlation between true T1 scores and the

## iMICs; values represent correlation coefficients

mn.imic2.set <- 1.25

## parameter controlling the mean of a 2nd threshold (between a "little

## improved" and "much improved";

## values are ratios of the mean iMIC2 to the SD of the PROM score at T1,

## reflecting the relative magnitude of the effect

sd.imic2.set <- 0.2

## parameter controlling the SD of the iMIC2 distribution

## values are proportions of the mean iMIC (i.e., sd.imic = sd.imic2)

cor.imic.imic2.set <- 0.75

## It is assumed that the thresholds are positively related.

## Mainly to ensure that the threshold between "little improved" and

## "much improved" is not smaller than the threshold between "unchanged"

## and "little improved".

ns <- sample.size.set

xt1 <- numeric(ns) # creates provisional true PROM measurement at T1

xt2 <- numeric(ns) # creates provisional true PROM measurement at T2

xtc <- numeric(ns) # creates provisional true change score variable

df <- data.frame(xt1, xt2, xtc) # creates dataframe

## --------------------------------

## Create a new simulated sample

# create the “true” PROM score at T1

( sd.xt1 <- sd.xt1.set * mn.xt1.set )

df$xt1 <- rnorm(n=ns, m=mn.xt1.set, s=sd.xt1)

# create iMIC variable that is correlated with the true T1 score

( mn.imic <- mn.imic.set*sd(df$xt1) )

( sd.imic <- sd.imic.set*mn.imic )

df$imic <- cor.t1.imic.set*df$xt1 + sqrt(1-cor.t1.imic.set^2)*

rnorm(ns,0,sd.xt1)

# Rescale the imic variable to the required sd and mean

df$imic <- df$imic - mean(df$imic)

df$imic <- df$imic * sd.imic / sd(df$imic)

df$imic <- df$imic + mn.imic

mean(df$imic)

sd(df$imic)

min(df$imic)

max(df$imic)

cor(df$imic,df$xt1)

# create iMIC2 variable that is correlated with iMIC

( mn.imic2 <- mn.imic2.set*sd(df$xt1) )

( sd.imic2 <- sd.imic2.set*mn.imic )

df$imic2 <- cor.imic.imic2.set*df$imic + sqrt(1-cor.imic.imic2.set^2)*

rnorm(ns,0,sd.imic2)

# Rescale the imic2 variable to the required sd and mean

df$imic2 <- df$imic2 - mean(df$imic2)

df$imic2 <- df$imic2 * sd.imic2 / sd(df$imic2)

df$imic2 <- df$imic2 + mn.imic2

mean(df$imic2)

sd(df$imic2)

cor(df$imic2,df$imic)

cor(df$imic2,df$xt1)

mean(df$imic2 - df$imic)

min(df$imic2 - df$imic) # this one should not be negative!

max(df$imic2 - df$imic)

# Create the "true" PROM change score that is correlated with the true T1 score

( sd.xtc <- sd.xtc.set * sd(df$xt1) )

mn.xtc <- 0

df$xtc <- cor.t1.xtc.set*df$xt1 + sqrt(1-cor.t1.xtc.set^2)*

rnorm(ns,0,sd.xtc)

# Rescale the change score to the required sd and mean

df$xtc <- df$xtc - mean(df$xtc)

df$xtc <- df$xtc * sd.xtc / sd(df$xtc)

df$xtc <- df$xtc + mean(df$imic) + mn.xtc.set * sd.xtc

cor(df$xt1,df$xtc)

# create "true" PROM score at T2

df$xt2 <- df$xt1 + df$xtc

# create GPC values (i.e., proportion improved according to the anchor)

# first, GPC based on the iMIC as benchmark

df$gpc1 <- numeric(ns)

df$gpc1[df$xtc > df$imic] <- 1

mean(df$gpc1)

df$gpc2 <- df$gpc1

df$gpc2[df$xtc > df$imic2] <- 0

mean(df$gpc2) # gpc2=1 is the subgroup "little improved"

table(df$gpc1, df$gpc2)

# create measurement error of the PROM measures at T1 and T2;

# add error to the true PROM scores at T1 and T2 and

# create (“observed”) PROM scores;

# round the T1 and T2 scores

sd.xt.error <- sqrt(((1-rr.xt.set)/rr.xt.set)*sd(df$xt1)^2)

error.t1 <- rnorm(n=ns, m=0, s=sd.xt.error)

error.t2 <- rnorm(n=ns, m=0, s=sd.xt.error)

df$xo1 <- round(df$xt1 + error.t1)

df$xo2 <- round(df$xt2 + error.t2)

# create the PROM change score with measurement error

df$xoc <- df$xo2 - df$xo1

# calculate and save the error components of the observed scores

df$xo1.err <- df$xo1 - df$xt1

df$xo2.err <- df$xo2 - df$xt2

df$xoc.err <- df$xoc - df$xtc

# calculate proportion improved and log-odds improvement

( q <- mean(df$gpc1) ) # q = proportion improvemed patients (gpc)

( pr <- log(q/(1-q)) ) # log-odds of improvement = log(pre-odds)

## Do logistic regression and calculate MIC(pred) and MIC(adjusted)

( cor.gpc.xoc <- cor(df$gpc1, df$xoc) )

mylogit <- glm(gpc1 ~ xoc, data = df, family = "binomial")

C <- coef(mylogit)[1] # intercept coefficient C

B <- coef(mylogit)[2] # regression coefficient B

( mic.pred <- (pr-C)/B ) # MIC(predicted)

cf <- 0.09 * sd(df$xoc) + 0.103 * sd(df$xoc) * cor.gpc.xoc

( mic.adj <- mic.pred - cf * pr )

## Do ROC analysis and calculate parameters en MIC(ROC)

rocobj <- roc(df$gpc1, df$xoc, quiet = TRUE)

( mic.roc <- coords(rocobj, x="best", input="threshold", ret="threshold",

best.method="youden", transpose = TRUE) )

## Mean change MIC method

( mic.mean <- mean(df[df$gpc2==1,]$xoc) )

## --------------------------------

# Results Table 1

mean(df$gpc1)

mean(df$gpc2)

mean(df$imic)

mean(df$imic2)

cor(df$xt1,df$xtc)

cor(df$xt1,df$imic)

mean(df$xo1)

mean(df$xo1.err)

mean(df$xo2)

mean(df$xo2.err)

mean(df$xoc)

mean(df$xoc.err)

mic.mean

mic.roc

mic.pred

mic.adj

################################################################

################################################################

##### MEDIAN-SPLIT SAMPLE ON BASELINE SCORE (METHOD 0)

median(df$xo1)

df1 <- df[df$xo1<median(df$xo1),] # low baseline group

df2 <- df[df$xo1>=median(df$xo1),] # high baseline group

ggplot(df1, aes(x=xo1)) +

geom_histogram(binwidth=2, colour="black", fill="white") +

dev.new(width=5, height=4) +

scale_x_continuous(limits=c(-2, 52), breaks=c(0,10,20,30,40,50))

# scale_y_continuous(limits=c(5, 15), breaks=c(5, 10, 15))

ggplot(df2, aes(x=xo1)) +

geom_histogram(binwidth=2, colour="black", fill="white") +

dev.new(width=5, height=4) +

scale_x_continuous(limits=c(48, 102), breaks=c(50,60,70,80,90,100))

# MICs low baseline group

( q1 <- mean(df1$gpc1) ) # q = proportion improvemed patients (gpc)

( pr1 <- log(q1/(1-q1)) ) # log-odds of improvement = log(pre-odds)

( cor.gpc.xoc.1 <- cor(df1$gpc1, df1$xoc) )

## Calculate MIC(pred) and MIC(adjusted) for df1

mylogit <- glm(gpc1 ~ xoc, data = df1, family = "binomial")

C <- coef(mylogit)[1] # intercept coefficient C

B <- coef(mylogit)[2] # regression coefficient B

( mic.pred.1 <- (pr1-C)/B ) # MIC(predicted)

cf <- 0.09 * sd(df1$xoc) + 0.103 * sd(df1$xoc) * cor.gpc.xoc.1

( mic.adj.1 <- mic.pred.1 - cf * pr1 )

## Calculate MIC(ROC) for df1

rocobj <- roc(df1$gpc1, df1$xoc, quiet = TRUE)

( mic.roc.1 <- coords(rocobj, x="best", input="threshold", ret="threshold",

best.method="youden", transpose = TRUE) )

## Mean change MIC for df1

( mic.mean.1 <- mean(df1[df1$gpc2==1,]$xoc) )

# MICs high baseline group

( q2 <- mean(df2$gpc1) ) # q = proportion improvemed patients (gpc)

( pr2 <- log(q2/(1-q2)) ) # log-odds of improvement = log(pre-odds)

( cor.gpc.xoc.2 <- cor(df2$gpc1, df2$xoc) )

## Calculate MIC(pred) and MIC(adjusted) for df2

mylogit <- glm(gpc1 ~ xoc, data = df2, family = "binomial")

C <- coef(mylogit)[1] # intercept coefficient C

B <- coef(mylogit)[2] # regression coefficient B

( mic.pred.2 <- (pr2-C)/B ) # MIC(predicted)

cf <- 0.09 * sd(df2$xoc) + 0.103 * sd(df2$xoc) * cor.gpc.xoc.2

( mic.adj.2 <- mic.pred.2 - cf * pr2 )

## Calculate MIC(ROC) for df2

rocobj <- roc(df2$gpc1, df2$xoc, quiet = TRUE)

( mic.roc.2 <- coords(rocobj, x="best", input="threshold", ret="threshold",

best.method="youden", transpose = TRUE) )

## Mean change MIC for df2

( mic.mean.2 <- mean(df2[df2$gpc2==1,]$xoc) )

# Table 1, column 2

mean(df1$gpc1)

mean(df1$gpc2)

mean(df1$imic)

mean(df1$imic2)

cor(df1$xt1,df1$xtc)

cor(df1$xt1,df1$imic)

mean(df1$xo1)

mean(df1$xo1.err)

mean(df1$xo2)

mean(df1$xo2.err)

mean(df1$xoc)

mean(df1$xoc.err)

mic.mean.1

mic.roc.1

mic.pred.1

mic.adj.1

# Table 1, column 3

mean(df2$gpc1)

mean(df2$gpc2)

mean(df2$imic)

mean(df2$imic2)

cor(df2$xt1,df2$xtc)

cor(df2$xt1,df2$imic)

mean(df2$xo1)

mean(df2$xo1.err)

mean(df2$xo2)

mean(df2$xo2.err)

mean(df2$xoc)

mean(df2$xoc.err)

mic.mean.2

mic.roc.2

mic.pred.2

mic.adj.2

###############################################################

############# RESAMPLING METHOD

dat <- df

## Low baseline group

mn.dat1 <- 41 # choose mean

sd.dat1 <- 10 # choose SD

data.frame(names(dat))

A <- 8 # column number of "xo1"

res.dist <- round( rnorm(n=0.5*nrow(dat), m=mn.dat1, s=sd.dat1) )

length(res.dist)

## Make a dataframe to hold the variables

cop <- matrix(NA, nrow=length(res.dist), ncol=ncol(dat))

colnames(cop) <- colnames(dat)

cop <- as.data.frame(cop)

head(cop)

## Fill cop$xo1 with the resample distribution scores

cop$xo1 <- res.dist

ggplot(cop, aes(x=xo1)) +

geom_histogram(binwidth=2, colour="black", fill="white") +

dev.new(width=5, height=4)

## Fill cop by sampling values from dat from min(dat) to max(dat)

## First, fill the lower half (< median(xo1))

for(sc in min(dat$xo1) : round(median(dat$xo1)) ) {

sset <- dat[dat$xo1==sc, -A] # source set of observations per score

for (i in 1:10) {

if (nrow(sset)==0) {

sset <- dat[dat$xo1==sc+i, -A] # if sc-category is empty, take next

}

}

if (nrow(cop[cop$xo1==sc,])>0) {

cop[cop$xo1==sc, -A] <- sset [ sample(1:dim(sset)[1],

size = nrow(cop[cop$xo1==sc,]), replace=TRUE),]

}

}

## Then, fill the upper half (> median(xo1))

for(sc in max(dat$xo1) : (round(median(dat$xo1))+1) ) {

sset <- dat[dat$xo1==sc, -A] # source set of observations per score

for (i in 1:10) {

if (nrow(sset)==0) {

sset <- dat[dat$xo1==sc-i, -A] # if sc-category is empty, take next

}

}

if (nrow(cop[cop$xo1==sc,])>0) {

cop[cop$xo1==sc, -A] <- sset [ sample(1:dim(sset)[1],

size = nrow(cop[cop$xo1==sc,]), replace=TRUE),]

}

}

## Impute the left tail (floor) of the xo1 distribution

sset <- dat[dat$xo1==min(dat$xo1), -A] # xo1 not imputed

cop[cop$xo1<min(dat$xo1), -A] <- sset [ sample(1:dim(sset)[1],

size = nrow(cop[cop$xo1<min(dat$xo1),]), replace=TRUE),]

## Impute the right tail (ceiling) of the xo1 distribution

sset <- dat[dat$xo1==max(dat$xo1), -A] # xo1 not imputed

cop[cop$xo1>max(dat$xo1), -A] <- sset [ sample(1:dim(sset)[1],

size = nrow(cop[cop$xo1>max(dat$xo1),]), replace=TRUE),]

summary(cop)

## Make a histogram of T1 score

ggplot(cop, aes(x=xo1)) +

geom_histogram(binwidth=2, colour="black", fill="white") +

dev.new(width=5, height=4)

##### CALCULATE MICs

( q <- mean(cop$gpc1) ) # q = proportion improvemed patients (gpc)

( pr <- log(q/(1-q)) ) # log-odds of improvement = log(pre-odds)

( cor.gpc.xoc <- cor(cop$gpc1, cop$xoc) )

## Calculate MIC(pred) and MIC(adjusted) for cop

mylogit <- glm(gpc1 ~ xoc, data = cop, family = "binomial")

C <- coef(mylogit)[1] # intercept coefficient C

B <- coef(mylogit)[2] # regression coefficient B

( mic.pred <- (pr-C)/B ) # MIC(predicted)

cf <- 0.09 * sd(cop$xoc) + 0.103 * sd(cop$xoc) * cor.gpc.xoc

( mic.adj <- mic.pred - cf * pr )

## Calculate MIC(ROC) for cop

rocobj <- roc(cop$gpc1, cop$xoc, quiet = TRUE)

( mic.roc <- coords(rocobj, x="best", input="threshold", ret="threshold",

best.method="youden", transpose = TRUE) )

## Mean change MIC method

( mic.mean <- mean(cop[cop$gpc2==1,]$xoc) )

## Table 1, columns 4

mean(cop$gpc1)

mean(cop$gpc2)

mean(cop$imic)

mean(cop$imic2)

cor(cop$xt1,cop$xtc)

cor(cop$xt1,cop$imic)

mean(cop$xo1)

mean(cop$xo1.err)

mean(cop$xo2)

mean(cop$xo2.err)

mean(cop$xoc)

mean(cop$xoc.err)

mic.mean

mic.roc

mic.pred

mic.adj

############################################

############# High baseline group

mn.dat1 <- 59 # choose mean

sd.dat1 <- 10 # choose SD

data.frame(names(dat))

A <- 8 # column number of "xo1"

res.dist <- round( rnorm(n=0.5*nrow(dat), m=mn.dat1, s=sd.dat1) )

length(res.dist)

## Make a dataframe to hold the variables

cop <- matrix(NA, nrow=length(res.dist), ncol=ncol(dat))

colnames(cop) <- colnames(dat)

cop <- as.data.frame(cop)

head(cop)

## Fill cop$xo1 with the resample distribution scores

cop$xo1 <- res.dist

ggplot(cop, aes(x=xo1)) +

geom_histogram(binwidth=2, colour="black", fill="white") +

dev.new(width=5, height=4)

## Fill cop by sampling values from dat from min(dat) to max(dat)

## First, fill the lower half (< median(xo1))

for(sc in min(dat$xo1) : round(median(dat$xo1)) ) {

sset <- dat[dat$xo1==sc, -A] # source set of observations per score

for (i in 1:10) {

if (nrow(sset)==0) {

sset <- dat[dat$xo1==sc+i, -A] # if sc-category is empty, take next

}

}

if (nrow(cop[cop$xo1==sc,])>0) {

cop[cop$xo1==sc, -A] <- sset [ sample(1:dim(sset)[1],

size = nrow(cop[cop$xo1==sc,]), replace=TRUE),]

}

}

## Then, fill the upper half (> median(xo1))

for(sc in max(dat$xo1) : (round(median(dat$xo1))+1) ) {

sset <- dat[dat$xo1==sc, -A] # source set of observations per score

for (i in 1:10) {

if (nrow(sset)==0) {

sset <- dat[dat$xo1==sc-i, -A] # if sc-category is empty, take next

}

}

if (nrow(cop[cop$xo1==sc,])>0) {

cop[cop$xo1==sc, -A] <- sset [ sample(1:dim(sset)[1],

size = nrow(cop[cop$xo1==sc,]), replace=TRUE),]

}

}

## Impute the left tail (floor) of the xo1 distribution

sset <- dat[dat$xo1==min(dat$xo1), -A] # xo1 not imputed

cop[cop$xo1<min(dat$xo1), -A] <- sset [ sample(1:dim(sset)[1],

size = nrow(cop[cop$xo1<min(dat$xo1),]), replace=TRUE),]

## Impute the right tail (ceiling) of the xo1 distribution

sset <- dat[dat$xo1==max(dat$xo1), -A] # xo1 not imputed

cop[cop$xo1>max(dat$xo1), -A] <- sset [ sample(1:dim(sset)[1],

size = nrow(cop[cop$xo1>max(dat$xo1),]), replace=TRUE),]

summary(cop)

## Make a histogram of T1 score

ggplot(cop, aes(x=xo1)) +

geom_histogram(binwidth=2, colour="black", fill="white") +

dev.new(width=5, height=4)

##### CALCULATE MICs

( q <- mean(cop$gpc1) ) # q = proportion improvemed patients (gpc)

( pr <- log(q/(1-q)) ) # log-odds of improvement = log(pre-odds)

( cor.gpc.xoc <- cor(cop$gpc1, cop$xoc) )

## Calculate MIC(pred) and MIC(adjusted) for cop

mylogit <- glm(gpc1 ~ xoc, data = cop, family = "binomial")

C <- coef(mylogit)[1] # intercept coefficient C

B <- coef(mylogit)[2] # regression coefficient B

( mic.pred <- (pr-C)/B ) # MIC(predicted)

cf <- 0.09 * sd(cop$xoc) + 0.103 * sd(cop$xoc) * cor.gpc.xoc

( mic.adj <- mic.pred - cf * pr )

## Calculate MIC(ROC) for cop

rocobj <- roc(cop$gpc1, cop$xoc, quiet = TRUE)

( mic.roc <- coords(rocobj, x="best", input="threshold", ret="threshold",

best.method="youden", transpose = TRUE) )

## Mean change MIC method

( mic.mean <- mean(cop[cop$gpc2==1,]$xoc) )

## Table 1, columns 5

mean(cop$gpc1)

mean(cop$gpc2)

mean(cop$imic)

mean(cop$imic2)

cor(cop$xt1,cop$xtc)

cor(cop$xt1,cop$imic)

mean(cop$xo1)

mean(cop$xo1.err)

mean(cop$xo2)

mean(cop$xo2.err)

mean(cop$xoc)

mean(cop$xoc.err)

mic.mean

mic.roc

mic.pred

mic.adj

###########################################################################

############################################################################

### ###

### SECTION 2.2. BASELINE DEPENDENCY SIMULATIONS TABLE 2 ###

### ###

############################################################################

library(pROC)

library(ggplot2)

library(psych)

rm(list=ls(all=TRUE))

############## WITHOUT PRESENT STATE BIAS ################

set.seed (12345)

## --------------------------------

## Set sample parameters

sample.size.set <- 100000

## parameter controlling the sample size

mn.xt1.set <- 50

## mean true HRQOL score at T1

sd.xt1.set <- 0.20

## parameter controlling the SD of the true HRQOL score at T1;

## values are ratios of the SD of the true HRQOL score to the mean

## of the true HRQOL score at T1

rr.xt.set <- 0.85

## reliability coefficient of HRQOL score (T1)

mn.imic.set <- 0.75

## parameter controlling the mean iMIC (= genuine MIC; gMIC);

## values are ratios of the mean iMIC to the SD of the HRQOL score at T1,

## reflecting the relative magnitude of the effect needed to qualify as

## genuine MIC (gMIC): 0.5 moderate, 0.8 large effect

sd.imic.set <- 0.2

## parameter controlling the SD of the iMICs

## values are proportions of the mean iMIC (or gMIC)

## TAKE CARE: the some iMIC values should not approach zero as this

## will not be realistic

sd.xtc.set <- 1

## parameter controlling the SD of the true HRQOL change score (T2-T1),

## i.e. variability of change;

## values are ratios of the SD of the true change score to the SD of the

## true HRQOL score at T1

mn.xtc.set <- 0

## parameter controlling the mean true HRQOL change score;

## values express the difference between the mean true change score and

## the gMIC in SD units of the true change score;

## note: the position of the mean true change score relative to the gMIC

## determines the PROPORTION IMPROVED

cor.t1.xtc.set <- 0

## parameter controlling the correlation between true T1 scores and the

## true change scores; values represent correlation coefficients

cor.t1.imic.set <- 0

## parameter controlling the correlation between true T1 scores and the

## iMICs; values represent correlation coefficients

mn.imic2.set <- 1.25

## parameter controlling the mean of a 2nd threshold (between a "little

## improved" and "much improved";

## values are ratios of the mean iMIC2 to the SD of the HRQOL score at T1,

## reflecting the relative magnitude of the effect

sd.imic2.set <- 0.2

## parameter controlling the SD of the iMIC2 distribution

## values are proportions of the mean iMIC (i.e., sd.imic = sd.imic2)

cor.imic.imic2.set <- 0.75

## It is assumed that the thresholds are positively related.

## Mainly to ensure that the threshold between "little improved" and

## "much improved" is not smaller than the threshold between "unchanged"

## and "little improved".

ns <- sample.size.set

xt1 <- numeric(ns) # creates provisional true HRQOL measurement at T1

xt2 <- numeric(ns) # creates provisional true HRQOL measurement at T2

xtc <- numeric(ns) # creates provisional true change score variable

df <- data.frame(xt1, xt2, xtc) # creates dataframe

## --------------------------------

## Create a new simulated sample

# create the “true” HRQOL score at T1

( sd.xt1 <- sd.xt1.set * mn.xt1.set )

df$xt1 <- rnorm(n=ns, m=mn.xt1.set, s=sd.xt1)

# create iMIC variable that is correlated with the true T1 score

( mn.imic <- mn.imic.set*sd(df$xt1) )

( sd.imic <- sd.imic.set*mn.imic )

df$imic <- cor.t1.imic.set*df$xt1 + sqrt(1-cor.t1.imic.set^2)*

rnorm(ns,0,sd.xt1)

# Rescale the imic variable to the required sd and mean

df$imic <- df$imic - mean(df$imic)

df$imic <- df$imic * sd.imic / sd(df$imic)

df$imic <- df$imic + mn.imic

mean(df$imic)

min(df$imic)

max(df$imic)

sd(df$imic)

cor(df$imic,df$xt1)

# create iMIC2 variable that is correlated with iMIC

( mn.imic2 <- mn.imic2.set*sd(df$xt1) )

( sd.imic2 <- sd.imic2.set*mn.imic )

df$imic2 <- cor.imic.imic2.set*df$imic + sqrt(1-cor.imic.imic2.set^2)*

rnorm(ns,0,sd.imic2)

# Rescale the imic2 variable to the required sd and mean

df$imic2 <- df$imic2 - mean(df$imic2)

df$imic2 <- df$imic2 * sd.imic2 / sd(df$imic2)

df$imic2 <- df$imic2 + mn.imic2

mean(df$imic2)

sd(df$imic2)

cor(df$imic2,df$imic)

cor(df$imic2,df$xt1)

mean(df$imic2 - df$imic)

min(df$imic2 - df$imic) # this one should not be negative!

max(df$imic2 - df$imic)

# Create the "true" HRQOL change score that is correlated with the true T1 score

( sd.xtc <- sd.xtc.set * sd(df$xt1) )

mn.xtc <- 0

df$xtc <- cor.t1.xtc.set*df$xt1 + sqrt(1-cor.t1.xtc.set^2)*

rnorm(ns,0,sd.xtc)

# Rescale the change score to the required sd and mean

df$xtc <- df$xtc - mean(df$xtc)

df$xtc <- df$xtc * sd.xtc / sd(df$xtc)

df$xtc <- df$xtc + mean(df$imic) + mn.xtc.set * sd.xtc

cor(df$xt1,df$xtc)

# create "true" HRQOL score at T2

df$xt2 <- df$xt1 + df$xtc

# create GPC values (i.e., proportion improved according to the anchor)

# first, GPC based on the iMIC as benchmark

df$gpc1 <- numeric(ns)

df$gpc1[df$xtc > df$imic] <- 1

mean(df$gpc1)

df$gpc2 <- df$gpc1

df$gpc2[df$xtc > df$imic2] <- 0

mean(df$gpc2) # gpc2=1 is the subgroup "little improved"

table(df$gpc1, df$gpc2)

# create measurement error of the HRQOL measures at T1 and T2;

# add error to the true HRQOL scores at T1 and T2 and

# create (“observed”) HRQOL scores;

# round the T1 and T2 scores

sd.xt.error <- sqrt(((1-rr.xt.set)/rr.xt.set)*sd(df$xt1)^2)

error.t1 <- rnorm(n=ns, m=0, s=sd.xt.error)

error.t2 <- rnorm(n=ns, m=0, s=sd.xt.error)

df$xo1 <- round(df$xt1 + error.t1)

df$xo2 <- round(df$xt2 + error.t2)

# create the HRQOL change score with measurement error

df$xoc <- df$xo2 - df$xo1

# calculate and save the error components of the observed scores

df$xo1.err <- df$xo1 - df$xt1

df$xo2.err <- df$xo2 - df$xt2

df$xoc.err <- df$xoc - df$xtc

# calculate proportion improved and log-odds improvement

( q <- mean(df$gpc1) ) # q = proportion improvemed patients (gpc)

( pr <- log(q/(1-q)) ) # log-odds of improvement = log(pre-odds)

## Do logistic regression and calculate MIC(pred) and MIC(adjusted)

( cor.gpc.xoc <- cor(df$gpc1, df$xoc) )

mylogit <- glm(gpc1 ~ xoc, data = df, family = "binomial")

C <- coef(mylogit)[1] # intercept coefficient C

B <- coef(mylogit)[2] # regression coefficient B

( mic.pred <- (pr-C)/B ) # MIC(predicted)

cf <- 0.09 * sd(df$xoc) + 0.103 * sd(df$xoc) * cor.gpc.xoc

( mic.adj <- mic.pred - cf * pr )

## Do ROC analysis and calculate parameters en MIC(ROC)

rocobj <- roc(df$gpc1, df$xoc, quiet = TRUE)

( mic.roc <- coords(rocobj, x="best", input="threshold", ret="threshold",

best.method="youden", transpose = TRUE) )

## Mean change MIC method

( mic.mean <- mean(df[df$gpc2==1,]$xoc) )

## --------------------------------

## Results Table 1, column 1

mean(df$gpc1)

mean(df$gpc2)

mean(df$imic)

mean(df$imic2)

cor(df$xt1,df$xtc)

cor(df$xt1,df$imic)

mean(df$xo1)

mean(df$xo1.err)

mean(df$xo2)

mean(df$xo2.err)

mean(df$xoc)

mean(df$xoc.err)

mic.mean

mic.roc

mic.pred

mic.adj

################################################################

#### Create independent correlated measurement

cor.xt1.yt1 <- 0.83

df$yt1 <- cor.xt1.yt1*df$xt1 + sqrt(1-cor.xt1.yt1^2)*rnorm(ns,0,10)

cor(df$yt1,df$xt1)

rr.xt.set # reliability of observed score

sd.yt.error <- sqrt(((1-rr.xt.set)/rr.xt.set)*sd(df$yt1)^2)

error.yt1 <- rnorm(n=ns, m=0, s=sd.yt.error)

df$yo1 <- round(df$yt1 + error.yt1)

cor(df$xo1, df$yo1) # correlation between observed baseline variables

##### MEDIAN-SPLIT SAMPLE ON BASELINE SCORE OF ANOTHER SCALE (METHOD 2)

median(df$yo1)

df1 <- df[df$yo1<median(df$yo1),] # low baseline group

df2 <- df[df$yo1>=median(df$yo1),] # high baseline group

ggplot(df1, aes(x=xo1)) +

geom_histogram(binwidth=2, colour="black", fill="white") +

dev.new(width=5, height=4) +

scale_x_continuous(limits=c(0, 90), breaks=c(0,20,40,60,80))

# scale_y_continuous(limits=c(5, 15), breaks=c(5, 10, 15))

ggplot(df2, aes(x=xo1)) +

geom_histogram(binwidth=2, colour="black", fill="white") +

dev.new(width=5, height=4) +

scale_x_continuous(limits=c(15, 100), breaks=c(20,40,60,80))

# MICs low baseline group

( q1 <- mean(df1$gpc1) ) # q = proportion improvemed patients (gpc)

( pr1 <- log(q1/(1-q1)) ) # log-odds of improvement = log(pre-odds)

( cor.gpc.xoc.1 <- cor(df1$gpc1, df1$xoc) )

## Calculate MIC(pred) and MIC(adjusted) for df1

mylogit <- glm(gpc1 ~ xoc, data = df1, family = "binomial")

C <- coef(mylogit)[1] # intercept coefficient C

B <- coef(mylogit)[2] # regression coefficient B

( mic.pred.1 <- (pr1-C)/B ) # MIC(predicted)

cf <- 0.09 * sd(df1$xoc) + 0.103 * sd(df1$xoc) * cor.gpc.xoc.1

( mic.adj.1 <- mic.pred.1 - cf * pr1 )

## Calculate MIC(ROC) for df1

rocobj <- roc(df1$gpc1, df1$xoc, quiet = TRUE)

( mic.roc.1 <- coords(rocobj, x="best", input="threshold", ret="threshold",

best.method="youden", transpose = TRUE) )

## Mean change MIC for df1

( mic.mean.1 <- mean(df1[df1$gpc2==1,]$xoc) )

# MICs high baseline group

( q2 <- mean(df2$gpc1) ) # q = proportion improvemed patients (gpc)

( pr2 <- log(q2/(1-q2)) ) # log-odds of improvement = log(pre-odds)

( cor.gpc.xoc.2 <- cor(df2$gpc1, df2$xoc) )

## Calculate MIC(pred) and MIC(adjusted) for df2

mylogit <- glm(gpc1 ~ xoc, data = df2, family = "binomial")

C <- coef(mylogit)[1] # intercept coefficient C

B <- coef(mylogit)[2] # regression coefficient B

( mic.pred.2 <- (pr2-C)/B ) # MIC(predicted)

cf <- 0.09 * sd(df2$xoc) + 0.103 * sd(df2$xoc) * cor.gpc.xoc.2

( mic.adj.2 <- mic.pred.2 - cf * pr2 )

## Calculate MIC(ROC) for df2

rocobj <- roc(df2$gpc1, df2$xoc, quiet = TRUE)

( mic.roc.2 <- coords(rocobj, x="best", input="threshold", ret="threshold",

best.method="youden", transpose = TRUE) )

## Mean change MIC for df2

( mic.mean.2 <- mean(df2[df2$gpc2==1,]$xoc) )

# Table 2, column 2

mean(df1$gpc1)

mean(df1$gpc2)

mean(df1$imic)

mean(df1$imic2)

cor(df1$xt1,df1$xtc)

cor(df1$xt1,df1$imic)

mean(df1$xo1)

mean(df1$xo1.err)

mean(df1$xo2)

mean(df1$xo2.err)

mean(df1$xoc)

mean(df1$xoc.err)

mic.mean.1

mic.roc.1

mic.pred.1

mic.adj.1

# Table 2, column 3

mean(df2$gpc1)

mean(df2$gpc2)

mean(df2$imic)

mean(df2$imic2)

cor(df2$xt1,df2$xtc)

cor(df2$xt1,df2$imic)

mean(df2$xo1)

mean(df2$xo1.err)

mean(df2$xo2)

mean(df2$xo2.err)

mean(df2$xoc)

mean(df2$xoc.err)

mic.mean.2

mic.roc.2

mic.pred.2

mic.adj.2

################################################################

################################################################

############### WITH PRESENT STATE BIAS ################

rm(list=ls(all=TRUE))

set.seed (12345)

## --------------------------------

## Set sample parameters

sample.size.set <- 100000

## parameter controlling the sample size

mn.xt1.set <- 50

## mean true HRQOL score at T1

sd.xt1.set <- 0.20

## parameter controlling the SD of the true HRQOL score at T1;

## values are ratios of the SD of the true HRQOL score to the mean

## of the true HRQOL score at T1

rr.xt.set <- 0.85

## reliability coefficient of HRQOL score (T1)

mn.imic.set <- 0.75

## parameter controlling the mean iMIC (= genuine MIC; gMIC);

## values are ratios of the mean iMIC to the SD of the HRQOL score at T1,

## reflecting the relative magnitude of the effect needed to qualify as

## genuine MIC (gMIC): 0.5 moderate, 0.8 large effect

sd.imic.set <- 0.2

## parameter controlling the SD of the iMICs

## values are proportions of the mean iMIC (or gMIC)

## TAKE CARE: the some iMIC values should not approach zero as this

## will not be realistic

sd.xtc.set <- 1

## parameter controlling the SD of the true HRQOL change score (T2-T1),

## i.e. variability of change;

## values are ratios of the SD of the true change score to the SD of the

## true HRQOL score at T1

mn.xtc.set <- 0

## parameter controlling the mean true HRQOL change score;

## values express the difference between the mean true change score and

## the gMIC in SD units of the true change score;

## note: the position of the mean true change score relative to the gMIC

## determines the PROPORTION IMPROVED

cor.t1.xtc.set <- -0.13 # SMALL NEG. COR. TO PREVENT PI NOT 0.5

## parameter controlling the correlation between true T1 scores and the

## true change scores; values represent correlation coefficients

cor.t1.imic.set <- -0.8 # CREATES BASELINE DEPENDENCY OF MIC

## parameter controlling the correlation between true T1 scores and the

## iMICs; values represent correlation coefficients

mn.imic2.set <- 1.25

## parameter controlling the mean of a 2nd threshold (between a "little

## improved" and "much improved";

## values are ratios of the mean iMIC2 to the SD of the HRQOL score at T1,

## reflecting the relative magnitude of the effect

sd.imic2.set <- 0.2

## parameter controlling the SD of the iMIC2 distribution

## values are proportions of the mean iMIC (i.e., sd.imic = sd.imic2)

cor.imic.imic2.set <- 0.75

## It is assumed that the thresholds are positively related.

## Mainly to ensure that the threshold between "little improved" and

## "much improved" is not smaller than the threshold between "unchanged"

## and "little improved".

ns <- sample.size.set

xt1 <- numeric(ns) # creates provisional true HRQOL measurement at T1

xt2 <- numeric(ns) # creates provisional true HRQOL measurement at T2

xtc <- numeric(ns) # creates provisional true change score variable

df <- data.frame(xt1, xt2, xtc) # creates dataframe

## --------------------------------

## Create a new simulated sample

# create the “true” HRQOL score at T1

( sd.xt1 <- sd.xt1.set * mn.xt1.set )

df$xt1 <- rnorm(n=ns, m=mn.xt1.set, s=sd.xt1)

# create iMIC variable that is correlated with the true T1 score

( mn.imic <- mn.imic.set*sd(df$xt1) )

( sd.imic <- sd.imic.set*mn.imic )

df$imic <- cor.t1.imic.set*df$xt1 + sqrt(1-cor.t1.imic.set^2)*

rnorm(ns,0,sd.xt1)

# Rescale the imic variable to the required sd and mean

df$imic <- df$imic - mean(df$imic)

df$imic <- df$imic * sd.imic / sd(df$imic)

df$imic <- df$imic + mn.imic

mean(df$imic)

min(df$imic)

max(df$imic)

sd(df$imic)

cor(df$imic,df$xt1)

# create iMIC2 variable that is correlated with iMIC

( mn.imic2 <- mn.imic2.set*sd(df$xt1) )

( sd.imic2 <- sd.imic2.set*mn.imic )

df$imic2 <- cor.imic.imic2.set*df$imic + sqrt(1-cor.imic.imic2.set^2)*

rnorm(ns,0,sd.imic2)

# Rescale the imic2 variable to the required sd and mean

df$imic2 <- df$imic2 - mean(df$imic2)

df$imic2 <- df$imic2 * sd.imic2 / sd(df$imic2)

df$imic2 <- df$imic2 + mn.imic2

mean(df$imic2)

sd(df$imic2)

cor(df$imic2,df$imic)

cor(df$imic2,df$xt1)

mean(df$imic2 - df$imic)

min(df$imic2 - df$imic) # this one should not be negative!

max(df$imic2 - df$imic)

# Create the "true" HRQOL change score that is correlated with the true T1 score

( sd.xtc <- sd.xtc.set * sd(df$xt1) )

mn.xtc <- 0

df$xtc <- cor.t1.xtc.set*df$xt1 + sqrt(1-cor.t1.xtc.set^2)*

rnorm(ns,0,sd.xtc)

# Rescale the change score to the required sd and mean

df$xtc <- df$xtc - mean(df$xtc)

df$xtc <- df$xtc * sd.xtc / sd(df$xtc)

df$xtc <- df$xtc + mean(df$imic) + mn.xtc.set * sd.xtc

cor(df$xt1,df$xtc)

# create "true" HRQOL score at T2

df$xt2 <- df$xt1 + df$xtc

# create GPC values (i.e., proportion improved according to the anchor)

# first, GPC based on the iMIC as benchmark

df$gpc1 <- numeric(ns)

df$gpc1[df$xtc > df$imic] <- 1

mean(df$gpc1)

df$gpc2 <- df$gpc1

df$gpc2[df$xtc > df$imic2] <- 0

mean(df$gpc2) # gpc2=1 is the subgroup "little improved"

table(df$gpc1, df$gpc2)

# create measurement error of the HRQOL measures at T1 and T2;

# add error to the true HRQOL scores at T1 and T2 and

# create (“observed”) HRQOL scores;

# round the T1 and T2 scores

sd.xt.error <- sqrt(((1-rr.xt.set)/rr.xt.set)*sd(df$xt1)^2)

error.t1 <- rnorm(n=ns, m=0, s=sd.xt.error)

error.t2 <- rnorm(n=ns, m=0, s=sd.xt.error)

df$xo1 <- round(df$xt1 + error.t1)

df$xo2 <- round(df$xt2 + error.t2)

# create the HRQOL change score with measurement error

df$xoc <- df$xo2 - df$xo1

# calculate and save the error components of the observed scores

df$xo1.err <- df$xo1 - df$xt1

df$xo2.err <- df$xo2 - df$xt2

df$xoc.err <- df$xoc - df$xtc

# calculate proportion improved and log-odds improvement

( q <- mean(df$gpc1) ) # q = proportion improvemed patients (gpc)

( pr <- log(q/(1-q)) ) # log-odds of improvement = log(pre-odds)

## Do logistic regression and calculate MIC(pred) and MIC(adjusted)

( cor.gpc.xoc <- cor(df$gpc1, df$xoc) )

mylogit <- glm(gpc1 ~ xoc, data = df, family = "binomial")

C <- coef(mylogit)[1] # intercept coefficient C

B <- coef(mylogit)[2] # regression coefficient B

( mic.pred <- (pr-C)/B ) # MIC(predicted)

cf <- 0.09 * sd(df$xoc) + 0.103 * sd(df$xoc) * cor.gpc.xoc

( mic.adj <- mic.pred - cf * pr )

## Do ROC analysis and calculate parameters en MIC(ROC)

rocobj <- roc(df$gpc1, df$xoc, quiet = TRUE)

( mic.roc <- coords(rocobj, x="best", input="threshold", ret="threshold",

best.method="youden", transpose = TRUE) )

## Mean change MIC method

( mic.mean <- mean(df[df$gpc2==1,]$xoc) )

## --------------------------------

## Results Table 2, column 4

mean(df$gpc1)

mean(df$gpc2)

mean(df$imic)

mean(df$imic2)

cor(df$xt1,df$xtc)

cor(df$xt1,df$imic)

mean(df$xo1)

mean(df$xo1.err)

mean(df$xo2)

mean(df$xo2.err)

mean(df$xoc)

mean(df$xoc.err)

mic.mean

mic.roc

mic.pred

mic.adj

################################################################

#### Create independent correlated measurement

cor.xt1.yt1 <- 0.83

df$yt1 <- cor.xt1.yt1*df$xt1 + sqrt(1-cor.xt1.yt1^2)*rnorm(ns,0,10)

cor(df$yt1,df$xt1)

rr.xt.set # reliability of observed score

sd.yt.error <- sqrt(((1-rr.xt.set)/rr.xt.set)*sd(df$yt1)^2)

error.yt1 <- rnorm(n=ns, m=0, s=sd.yt.error)

df$yo1 <- round(df$yt1 + error.yt1)

cor(df$xo1, df$yo1) # correlation between observed baseline variables

##### MEDIAN-SPLIT SAMPLE ON BASELINE SCORE OF ANOTHER SCALE (METHOD 2)

median(df$yo1)

df1 <- df[df$yo1<median(df$yo1),] # low baseline group

df2 <- df[df$yo1>=median(df$yo1),] # high baseline group

ggplot(df1, aes(x=xo1)) +

geom_histogram(binwidth=2, colour="black", fill="white") +

dev.new(width=5, height=4) +

scale_x_continuous(limits=c(0, 90), breaks=c(0,20,40,60,80))

# scale_y_continuous(limits=c(5, 15), breaks=c(5, 10, 15))

ggplot(df2, aes(x=xo1)) +

geom_histogram(binwidth=2, colour="black", fill="white") +

dev.new(width=5, height=4) +

scale_x_continuous(limits=c(15, 100), breaks=c(20,40,60,80))

# MICs low baseline group

( q1 <- mean(df1$gpc1) ) # q = proportion improvemed patients (gpc)

( pr1 <- log(q1/(1-q1)) ) # log-odds of improvement = log(pre-odds)

( cor.gpc.xoc.1 <- cor(df1$gpc1, df1$xoc) )

## Calculate MIC(pred) and MIC(adjusted) for df1

mylogit <- glm(gpc1 ~ xoc, data = df1, family = "binomial")

C <- coef(mylogit)[1] # intercept coefficient C

B <- coef(mylogit)[2] # regression coefficient B

( mic.pred.1 <- (pr1-C)/B ) # MIC(predicted)

cf <- 0.09 * sd(df1$xoc) + 0.103 * sd(df1$xoc) * cor.gpc.xoc.1

( mic.adj.1 <- mic.pred.1 - cf * pr1 )

## Calculate MIC(ROC) for df1

rocobj <- roc(df1$gpc1, df1$xoc, quiet = TRUE)

( mic.roc.1 <- coords(rocobj, x="best", input="threshold", ret="threshold",

best.method="youden", transpose = TRUE) )

## Mean change MIC for df1

( mic.mean.1 <- mean(df1[df1$gpc2==1,]$xoc) )

# MICs high baseline group

( q2 <- mean(df2$gpc1) ) # q = proportion improvemed patients (gpc)

( pr2 <- log(q2/(1-q2)) ) # log-odds of improvement = log(pre-odds)

( cor.gpc.xoc.2 <- cor(df2$gpc1, df2$xoc) )

## Calculate MIC(pred) and MIC(adjusted) for df2

mylogit <- glm(gpc1 ~ xoc, data = df2, family = "binomial")

C <- coef(mylogit)[1] # intercept coefficient C

B <- coef(mylogit)[2] # regression coefficient B

( mic.pred.2 <- (pr2-C)/B ) # MIC(predicted)

cf <- 0.09 * sd(df2$xoc) + 0.103 * sd(df2$xoc) * cor.gpc.xoc.2

( mic.adj.2 <- mic.pred.2 - cf * pr2 )

## Calculate MIC(ROC) for df2

rocobj <- roc(df2$gpc1, df2$xoc, quiet = TRUE)

( mic.roc.2 <- coords(rocobj, x="best", input="threshold", ret="threshold",

best.method="youden", transpose = TRUE) )

## Mean change MIC for df2

( mic.mean.2 <- mean(df2[df2$gpc2==1,]$xoc) )

# Table 2, column 5

mean(df1$gpc1)

mean(df1$gpc2)

mean(df1$imic)

mean(df1$imic2)

cor(df1$xt1,df1$xtc)

cor(df1$xt1,df1$imic)

mean(df1$xo1)

mean(df1$xo1.err)

mean(df1$xo2)

mean(df1$xo2.err)

mean(df1$xoc)

mean(df1$xoc.err)

mic.mean.1

mic.roc.1

mic.pred.1

mic.adj.1

# Table 2, column 6

mean(df2$gpc1)

mean(df2$gpc2)

mean(df2$imic)

mean(df2$imic2)

cor(df2$xt1,df2$xtc)

cor(df2$xt1,df2$imic)

mean(df2$xo1)

mean(df2$xo1.err)

mean(df2$xo2)

mean(df2$xo2.err)

mean(df2$xoc)

mean(df2$xoc.err)

mic.mean.2

mic.roc.2

mic.pred.2

mic.adj.2

####################################################################

############################################################################

### ###

### SECTION 2.3. BASELINE DEPENDENCY SIMULATIONS TABLE 3 ###

### WITHOUT BASELINE DEPENDENT MIC ###

### ###

############################################################################

library(pROC)

library(ggplot2)

library(psych)

rm(list=ls(all=TRUE))

############## WITHOUT PRESENT STATE BIAS ################

set.seed (12345)

## --------------------------------

## Set sample parameters

sample.size.set <- 100000

## parameter controlling the sample size

mn.xt1.set <- 50

## mean true HRQOL score at T1

sd.xt1.set <- 0.20

## parameter controlling the SD of the true HRQOL score at T1;

## values are ratios of the SD of the true HRQOL score to the mean

## of the true HRQOL score at T1

rr.xt.set <- 0.85

## reliability coefficient of HRQOL score (T1)

mn.imic.set <- 0.75

## parameter controlling the mean iMIC (= genuine MIC; gMIC);

## values are ratios of the mean iMIC to the SD of the HRQOL score at T1,

## reflecting the relative magnitude of the effect needed to qualify as

## genuine MIC (gMIC): 0.5 moderate, 0.8 large effect

sd.imic.set <- 0.2

## parameter controlling the SD of the iMICs

## values are proportions of the mean iMIC (or gMIC)

## TAKE CARE: the some iMIC values should not approach zero as this

## will not be realistic

sd.xtc.set <- 1

## parameter controlling the SD of the true HRQOL change score (T2-T1),

## i.e. variability of change;

## values are ratios of the SD of the true change score to the SD of the

## true HRQOL score at T1

mn.xtc.set <- 0

## parameter controlling the mean true HRQOL change score;

## values express the difference between the mean true change score and

## the gMIC in SD units of the true change score;

## note: the position of the mean true change score relative to the gMIC

## determines the PROPORTION IMPROVED

cor.t1.xtc.set <- 0

## parameter controlling the correlation between true T1 scores and the

## true change scores; values represent correlation coefficients

cor.t1.imic.set <- 0

## parameter controlling the correlation between true T1 scores and the

## iMICs; values represent correlation coefficients

mn.imic2.set <- 1.25

## parameter controlling the mean of a 2nd threshold (between a "little

## improved" and "much improved";

## values are ratios of the mean iMIC2 to the SD of the HRQOL score at T1,

## reflecting the relative magnitude of the effect

sd.imic2.set <- 0.2

## parameter controlling the SD of the iMIC2 distribution

## values are proportions of the mean iMIC (i.e., sd.imic = sd.imic2)

cor.imic.imic2.set <- 0.75

## It is assumed that the thresholds are positively related.

## Mainly to ensure that the threshold between "little improved" and

## "much improved" is not smaller than the threshold between "unchanged"

## and "little improved".

ns <- sample.size.set

xt1 <- numeric(ns) # creates provisional true HRQOL measurement at T1

xt2 <- numeric(ns) # creates provisional true HRQOL measurement at T2

xtc <- numeric(ns) # creates provisional true change score variable

df <- data.frame(xt1, xt2, xtc) # creates dataframe

## --------------------------------

## Create a new simulated sample

## Create parallel tests T1.PT1 and T1.PT2

# create "true" T1.PT1 and T1.PT2 scores

( sd.xt1.pt <- mn.xt1.set * sd.xt1.set / 2 )

df$xt1.pt1 <- rnorm(n=ns, m=mn.xt1.set/2, s=sd.xt1.pt)

df$xt1.pt2 <- df$xt1.pt1 # true T1 scores are the same

# create the “true” HRQOL scores at T1

df$xt1 <- df$xt1.pt1 + df$xt1.pt2

mean(df$xt1.pt1)

sd(df$xt1.pt1)

mean(df$xt1.pt2)

sd(df$xt1.pt2)

mean(df$xt1)

( sd.xt1 <- sd(df$xt1) )

# create iMIC variable that is correlated with the true T1 score

( mn.imic <- mn.imic.set*sd(df$xt1) )

( sd.imic <- sd.imic.set*mn.imic )

df$imic <- cor.t1.imic.set*df$xt1 + sqrt(1-cor.t1.imic.set^2)*

rnorm(ns,0,sd.xt1)

# Rescale the imic variable to the required sd and mean

df$imic <- df$imic - mean(df$imic)

df$imic <- df$imic * sd.imic / sd(df$imic)

df$imic <- df$imic + mn.imic

mean(df$imic)

min(df$imic)

max(df$imic)

sd(df$imic)

cor(df$imic,df$xt1)

# create iMIC2 variable that is correlated with iMIC

( mn.imic2 <- mn.imic2.set*sd(df$xt1) )

( sd.imic2 <- sd.imic2.set*mn.imic )

df$imic2 <- cor.imic.imic2.set*df$imic + sqrt(1-cor.imic.imic2.set^2)*

rnorm(ns,0,sd.imic2)

# Rescale the imic2 variable to the required sd and mean

df$imic2 <- df$imic2 - mean(df$imic2)

df$imic2 <- df$imic2 * sd.imic2 / sd(df$imic2)

df$imic2 <- df$imic2 + mn.imic2

mean(df$imic2)

sd(df$imic2)

cor(df$imic2,df$imic)

cor(df$imic2,df$xt1)

mean(df$imic2 - df$imic)

min(df$imic2 - df$imic) # this one should not be negative!

max(df$imic2 - df$imic)

# Create the "true" HRQOL change score that is correlated with the true T1 score

( sd.xtc.pt <- sd.xtc.set * sd(df$xt1) /2 )

df$xtc.pt1 <- cor.t1.xtc.set*df$xt1.pt1 + sqrt(1-cor.t1.xtc.set^2)*

rnorm(ns,0,sd.xtc.pt)

# Rescale the change score to the required sd and mean

df$xtc.pt1 <- df$xtc.pt1 - mean(df$xtc.pt1)

df$xtc.pt1 <- df$xtc.pt1 * sd.xtc.pt / sd(df$xtc.pt1)

df$xtc.pt1 <- df$xtc.pt1 + (mean(df$imic)/2) + (mn.xtc.set/2) * sd.xtc.pt

df$xtc.pt2 <- df$xtc.pt1 # true change scores are the same

cor(df$xt1.pt1,df$xtc.pt1)

cor(df$xt1.pt2,df$xtc.pt2)

# create true change score

df$xtc <- df$xtc.pt1 + df$xtc.pt2

mean(df$xtc.pt1)

sd(df$xtc.pt1)

mean(df$xtc.pt2)

sd(df$xtc.pt2)

mean(df$xtc)

sd(df$xtc)

cor(df$xt1,df$xtc)

# create "true" HRQOL score at T2

df$xt2.pt1 <- df$xt1.pt1 + df$xtc.pt1

df$xt2.pt2 <- df$xt2.pt1 # true T2 scores are the same

df$xt2 <- df$xt2.pt1 + df$xt2.pt2

mean(df$xt2.pt1)

sd(df$xt2.pt1)

mean(df$xt2.pt2)

sd(df$xt2.pt2)

mean(df$xt2)

sd(df$xt2)

# create GPC values (i.e., proportion improved according to the anchor)

# first, GPC based on the iMIC as benchmark

df$gpc1 <- numeric(ns)

df$gpc1[df$xtc > df$imic] <- 1

mean(df$gpc1)

df$gpc2 <- df$gpc1

df$gpc2[df$xtc > df$imic2] <- 0

mean(df$gpc2) # gpc2=1 is the subgroup "little improved"

table(df$gpc1, df$gpc2)

# create measurement error of the HRQOL measures at T1 and T2;

# add error to the true HRQOL scores at T1 and T2 and

# create (“observed”) HRQOL scores;

# round the T1 and T2 scores

sd.xt.error.pt <- (sqrt(((1-rr.xt.set)/rr.xt.set)*sd(df$xt1)^2))/sqrt(2)

error.t1.pt1 <- rnorm(n=ns, m=0, s=sd.xt.error.pt)

error.t1.pt2 <- rnorm(n=ns, m=0, s=sd.xt.error.pt)

error.t2.pt1 <- rnorm(n=ns, m=0, s=sd.xt.error.pt)

error.t2.pt2 <- rnorm(n=ns, m=0, s=sd.xt.error.pt)

df$xo1.pt1 <- round(df$xt1.pt1 + error.t1.pt1)

df$xo1.pt2 <- round(df$xt1.pt2 + error.t1.pt2)

df$xo2.pt1 <- round(df$xt2.pt1 + error.t2.pt1)

df$xo2.pt2 <- round(df$xt2.pt2 + error.t2.pt2)

df$xo1 <- df$xo1.pt1 + df$xo1.pt2

df$xo2 <- df$xo2.pt1 + df$xo2.pt2

var(df$xt1.pt1)/var(df$xo1.pt1) # reliability of parallel test 1 at T1

var(df$xt2.pt1)/var(df$xo2.pt1) # reliability of parallel test 1 at T2

var(df$xt1.pt2)/var(df$xo1.pt2) # reliability of parallel test 2 at T1

var(df$xt2.pt2)/var(df$xo2.pt2) # reliability of parallel test 2 at T2

var(df$xt1)/var(df$xo1) # reliability of total score T1

var(df$xt2)/var(df$xo2) # reliability of total score T2

# create the HRQOL change score with measurement error

df$xoc.pt1 <- df$xo2.pt1 - df$xo1.pt1

df$xoc.pt2 <- df$xo2.pt2 - df$xo1.pt2

df$xoc <- df$xo2 - df$xo1

# calculate and save the error components of the observed scores

df$xo1.err <- df$xo1 - df$xt1

df$xo2.err <- df$xo2 - df$xt2

df$xoc.err <- df$xoc - df$xtc

mean(df$xo1.err)

mean(df$xo2.err)

mean(df$xoc.err)

df$xo1.pt1.err <- df$xo1.pt1 - df$xt1.pt1

df$xo2.pt1.err <- df$xo2.pt1 - df$xt2.pt1

df$xoc.pt1.err <- df$xoc.pt1 - df$xtc.pt1

mean(df$xo1.pt1.err)

mean(df$xo2.pt1.err)

mean(df$xoc.pt1.err)

df$xo1.pt2.err <- df$xo1.pt2 - df$xt1.pt2

df$xo2.pt2.err <- df$xo2.pt2 - df$xt2.pt2

df$xoc.pt2.err <- df$xoc.pt2 - df$xtc.pt2

mean(df$xo1.pt2.err)

mean(df$xo2.pt2.err)

mean(df$xoc.pt2.err)

cor(df$xo1.pt1, df$xo1.pt2) # test-retest correlation of parallel test T1

# same as reliability of PT score at T1

# calculate proportion improved and log-odds improvement

( q <- mean(df$gpc1) ) # q = proportion improvemed patients (gpc)

( pr <- log(q/(1-q)) ) # log-odds of improvement = log(pre-odds)

## Do logistic regression and calculate MIC(pred) and MIC(adjusted)

( cor.gpc.xoc <- cor(df$gpc1, df$xoc) )

mylogit <- glm(gpc1 ~ xoc, data = df, family = "binomial")

C <- coef(mylogit)[1] # intercept coefficient C

B <- coef(mylogit)[2] # regression coefficient B

( mic.pred <- (pr-C)/B ) # MIC(predicted)

cf <- 0.09 * sd(df$xoc) + 0.103 * sd(df$xoc) * cor.gpc.xoc

( mic.adj <- mic.pred - cf * pr )

## Do ROC analysis and calculate parameters en MIC(ROC)

rocobj <- roc(df$gpc1, df$xoc, quiet = TRUE)

( mic.roc <- coords(rocobj, x="best", input="threshold", ret="threshold",

best.method="youden", transpose = TRUE) )

## Mean change MIC method

( mic.mean <- mean(df[df$gpc2==1,]$xoc) )

## --------------------------------

## Results Table 3, column 1

mean(df$gpc1)

mean(df$gpc2)

mean(df$imic)

mean(df$imic2)

cor(df$xt1,df$xtc)

cor(df$xt1,df$imic)

mean(df$xo1)

mean(df$xo1.err)

mean(df$xo2)

mean(df$xo2.err)

mean(df$xoc)

mean(df$xoc.err)

mic.mean

mic.roc

mic.pred

mic.adj

#########################################################################

## Median split dataset PT1 on baseline score of PT2

( med.coff <- median(df$xo1.pt2) )

df1 <- df[df$xo1.pt2 < med.coff,] # low baseline subgroup

df2 <- df[df$xo1.pt2 >= med.coff,] # high baseline subgroup

summary(df1$xo1.pt1)

summary(df2$xo1.pt2)

ggplot(df1, aes(x=xo1.pt1)) +

geom_histogram(binwidth=1, colour="black", fill="white") +

dev.new(width=5, height=4)

ggplot(df2, aes(x=xo1.pt1)) +

geom_histogram(binwidth=1, colour="black", fill="white") +

dev.new(width=5, height=4)

###### MICs low baseline group

( q1 <- mean(df1$gpc1) ) # q = proportion improvemed patients (gpc)

( pr1 <- log(q1/(1-q1)) ) # log-odds of improvement = log(pre-odds)

( cor.gpc.xoc.1 <- cor(df1$gpc1, df1$xoc.pt1) )

## Calculate MIC(pred) and MIC(adjusted) for df1

mylogit <- glm(gpc1 ~ xoc.pt1, data = df1, family = "binomial")

C <- coef(mylogit)[1] # intercept coefficient C

B <- coef(mylogit)[2] # regression coefficient B

( mic.pred.1 <- (pr1-C)/B ) # MIC(predicted)

cf <- 0.09 * sd(df1$xoc.pt1) + 0.103 * sd(df1$xoc.pt1) * cor.gpc.xoc.1

( mic.adj.1 <- mic.pred.1 - cf * pr1 )

## Calculate MIC(ROC) for df1

rocobj <- roc(df1$gpc1, df1$xoc.pt1, quiet = TRUE)

( mic.roc.1 <- coords(rocobj, x="best", input="threshold", ret="threshold",

best.method="youden", transpose = TRUE) )

## Mean change MIC method

( mic.mean.1 <- mean(df1[df1$gpc2==1,]$xoc.pt1) ) # mean change MIC group 1

###### MICs high baseline group

## Calculate MIC(pred) and MIC(adjusted) for df2

( q2 <- mean(df2$gpc1) ) # q = proportion improvemed patients (gpc)

( pr2 <- log(q2/(1-q2)) ) # log-odds of improvement = log(pre-odds)

( cor.gpc.xoc.2 <- cor(df2$gpc1, df2$xoc.pt2) )

mylogit <- glm(gpc1 ~ xoc.pt1, data = df2, family = "binomial")

C <- coef(mylogit)[1] # intercept coefficient C

B <- coef(mylogit)[2] # regression coefficient B

( mic.pred.2 <- (pr2-C)/B ) # MIC(predicted)

cf <- 0.09 * sd(df2$xoc.pt1) + 0.103 * sd(df2$xoc.pt1) * cor.gpc.xoc.2

( mic.adj.2 <- mic.pred.2 - cf * pr2 )

## Calculate MIC(ROC) for df2

rocobj <- roc(df2$gpc1, df2$xoc.pt1, quiet = TRUE)

( mic.roc.2 <- coords(rocobj, x="best", input="threshold", ret="threshold",

best.method="youden", transpose = TRUE) )

## Mean change MIC method

( mic.mean.2 <- mean(df2[df2$gpc2==1,]$xoc.pt1) ) # mean change MIC group 2

# Table 3, column 2

mean(df1$gpc1)

mean(df1$gpc2)

mean(df1$imic)

mean(df1$imic2)

cor(df1$xt1.pt1,df1$xtc.pt1)

cor(df1$xt1.pt1,df1$imic)

mean(df1$xo1.pt1)

mean(df1$xo1.pt1.err)

mean(df1$xo2.pt1)

mean(df1$xo2.pt1.err)

mean(df1$xoc.pt1)

mean(df1$xoc.pt1.err)

mic.mean.1

mic.roc.1

mic.pred.1

mic.adj.1

# Table 3, column 3

mean(df2$gpc1)

mean(df2$gpc2)

mean(df2$imic)

mean(df2$imic2)

cor(df2$xt1.pt1,df2$xtc.pt1)

cor(df2$xt1.pt1,df2$imic)

mean(df2$xo1.pt1)

mean(df2$xo1.pt1.err)

mean(df2$xo2.pt1)

mean(df2$xo2.pt1.err)

mean(df2$xoc.pt1)

mean(df2$xoc.pt1.err)

mic.mean.2

mic.roc.2

mic.pred.2

mic.adj.2

################################################################

## Median split dataset PT2 on baseline score of PT1

( med.coff <- median(df$xo1.pt1) )

df1 <- df[df$xo1.pt1 < med.coff,] # low baseline subgroup

df2 <- df[df$xo1.pt1 >= med.coff,] # high baseline subgroup

summary(df1$xo1.pt2)

summary(df2$xo1.pt2)

ggplot(df1, aes(x=xo1.pt2)) +

geom_histogram(binwidth=1, colour="black", fill="white") +

dev.new(width=5, height=4)

ggplot(df2, aes(x=xo1.pt2)) +

geom_histogram(binwidth=1, colour="black", fill="white") +

dev.new(width=5, height=4)

###### MICs low baseline group

## Calculate MIC(pred) and MIC(adjusted) for df1

( q1 <- mean(df1$gpc1) ) # q = proportion improvemed patients (gpc)

( pr1 <- log(q1/(1-q1)) ) # log-odds of improvement = log(pre-odds)

( cor.gpc.xoc.1 <- cor(df1$gpc1, df1$xoc.pt2) )

mylogit <- glm(gpc1 ~ xoc.pt2, data = df1, family = "binomial")

C <- coef(mylogit)[1] # intercept coefficient C

B <- coef(mylogit)[2] # regression coefficient B

( mic.pred.1 <- (pr1-C)/B ) # MIC(predicted)

cf <- 0.09 * sd(df1$xoc.pt2) + 0.103 * sd(df1$xoc.pt2) * cor.gpc.xoc.1

( mic.adj.1 <- mic.pred.1 - cf * pr1 )

## Calculate MIC(ROC) for df1

rocobj <- roc(df1$gpc1, df1$xoc.pt2, quiet = TRUE)

( mic.roc.1 <- coords(rocobj, x="best", input="threshold", ret="threshold",

best.method="youden", transpose = TRUE) )

## Mean change MIC method

( mic.mean.1 <- mean(df1[df1$gpc2==1,]$xoc.pt2) ) # mean change MIC group 1

###### MICs high baseline group

## Calculate MIC(pred) and MIC(adjusted) for df2

( q2 <- mean(df2$gpc1) ) # q = proportion improvemed patients (gpc)

( pr2 <- log(q2/(1-q2)) ) # log-odds of improvement = log(pre-odds)

( cor.gpc.xoc.2 <- cor(df2$gpc1, df2$xoc.pt1) )

mylogit <- glm(gpc1 ~ xoc.pt2, data = df2, family = "binomial")

C <- coef(mylogit)[1] # intercept coefficient C

B <- coef(mylogit)[2] # regression coefficient B

( mic.pred.2 <- (pr2-C)/B ) # MIC(predicted)

cf <- 0.09 * sd(df2$xoc.pt2) + 0.103 * sd(df2$xoc.pt2) * cor.gpc.xoc.2

( mic.adj.2 <- mic.pred.2 - cf * pr2 )

## Calculate MIC(ROC) for df2

rocobj <- roc(df2$gpc1, df2$xoc.pt2, quiet = TRUE)

( mic.roc.2 <- coords(rocobj, x="best", input="threshold", ret="threshold",

best.method="youden", transpose = TRUE) )

## Mean change MIC method

mean(df2[df2$gpc2==1,]$xoc.pt2) # mean change MIC group 2

# Table 3, column 4

mean(df1$gpc1)

mean(df1$gpc2)

mean(df1$imic)

mean(df1$imic2)

cor(df1$xt1.pt2,df1$xtc.pt2)

cor(df1$xt1.pt2,df1$imic)

mean(df1$xo1.pt2)

mean(df1$xo1.pt2.err)

mean(df1$xo2.pt2)

mean(df1$xo2.pt2.err)

mean(df1$xoc.pt2)

mean(df1$xoc.pt2.err)

mic.mean.1

mic.roc.1

mic.pred.1

mic.adj.1

# Table 3, column 5

mean(df2$gpc1)

mean(df2$gpc2)

mean(df2$imic)

mean(df2$imic2)

cor(df2$xt1.pt2,df2$xtc.pt2)

cor(df2$xt1.pt2,df2$imic)

mean(df2$xo1.pt2)

mean(df2$xo1.pt2.err)

mean(df2$xo2.pt2)

mean(df2$xo2.pt2.err)

mean(df2$xoc.pt2)

mean(df2$xoc.pt2.err)

mic.mean.2

mic.roc.2

mic.pred.2

mic.adj.2

################################################################

############################################################################

### ###

### SECTION 2.4. BASELINE DEPENDENCY SIMULATIONS TABLE 3 ###

### WITH BASELINE DEPENDENT MIC ###

### ###

############################################################################

library(pROC)

library(ggplot2)

library(psych)

rm(list=ls(all=TRUE))

############## WITH PRESENT STATE BIAS ################

set.seed (12345)

## --------------------------------

## Set sample parameters

sample.size.set <- 100000

## parameter controlling the sample size

mn.xt1.set <- 50

## mean true HRQOL score at T1

sd.xt1.set <- 0.20

## parameter controlling the SD of the true HRQOL score at T1;

## values are ratios of the SD of the true HRQOL score to the mean

## of the true HRQOL score at T1

rr.xt.set <- 0.85

## reliability coefficient of HRQOL score (T1)

mn.imic.set <- 0.75

## parameter controlling the mean iMIC (= genuine MIC; gMIC);

## values are ratios of the mean iMIC to the SD of the HRQOL score at T1,

## reflecting the relative magnitude of the effect needed to qualify as

## genuine MIC (gMIC): 0.5 moderate, 0.8 large effect

sd.imic.set <- 0.2

## parameter controlling the SD of the iMICs

## values are proportions of the mean iMIC (or gMIC)

## TAKE CARE: the some iMIC values should not approach zero as this

## will not be realistic

sd.xtc.set <- 1

## parameter controlling the SD of the true HRQOL change score (T2-T1),

## i.e. variability of change;

## values are ratios of the SD of the true change score to the SD of the

## true HRQOL score at T1

mn.xtc.set <- 0

## parameter controlling the mean true HRQOL change score;

## values express the difference between the mean true change score and

## the gMIC in SD units of the true change score;

## note: the position of the mean true change score relative to the gMIC

## determines the PROPORTION IMPROVED

cor.t1.xtc.set <- -0.13 # SMALL NEG. COR. TO PREVENT PI NOT 0.5

## parameter controlling the correlation between true T1 scores and the

## true change scores; values represent correlation coefficients

cor.t1.imic.set <- -0.8 # CREATES BASELINE DEPENDENCY OF MIC

## parameter controlling the correlation between true T1 scores and the

## iMICs; values represent correlation coefficients

mn.imic2.set <- 1.25

## parameter controlling the mean of a 2nd threshold (between a "little

## improved" and "much improved";

## values are ratios of the mean iMIC2 to the SD of the HRQOL score at T1,

## reflecting the relative magnitude of the effect

sd.imic2.set <- 0.2

## parameter controlling the SD of the iMIC2 distribution

## values are proportions of the mean iMIC (i.e., sd.imic = sd.imic2)

cor.imic.imic2.set <- 0.75

## It is assumed that the thresholds are positively related.

## Mainly to ensure that the threshold between "little improved" and

## "much improved" is not smaller than the threshold between "unchanged"

## and "little improved".

ns <- sample.size.set

xt1 <- numeric(ns) # creates provisional true HRQOL measurement at T1

xt2 <- numeric(ns) # creates provisional true HRQOL measurement at T2

xtc <- numeric(ns) # creates provisional true change score variable

df <- data.frame(xt1, xt2, xtc) # creates dataframe

## --------------------------------

## Create a new simulated sample

## Create parallel tests T1.PT1 and T1.PT2

# create "true" T1.PT1 and T1.PT2 scores

( sd.xt1.pt <- mn.xt1.set * sd.xt1.set / 2 )

df$xt1.pt1 <- rnorm(n=ns, m=mn.xt1.set/2, s=sd.xt1.pt)

df$xt1.pt2 <- df$xt1.pt1 # true T1 scores are the same

# create the “true” HRQOL scores at T1

df$xt1 <- df$xt1.pt1 + df$xt1.pt2

mean(df$xt1.pt1)

sd(df$xt1.pt1)

mean(df$xt1.pt2)

sd(df$xt1.pt2)

mean(df$xt1)

( sd.xt1 <- sd(df$xt1) )

# create iMIC variable that is correlated with the true T1 score

( mn.imic <- mn.imic.set*sd(df$xt1) )

( sd.imic <- sd.imic.set*mn.imic )

df$imic <- cor.t1.imic.set*df$xt1 + sqrt(1-cor.t1.imic.set^2)*

rnorm(ns,0,sd.xt1)

# Rescale the imic variable to the required sd and mean

df$imic <- df$imic - mean(df$imic)

df$imic <- df$imic * sd.imic / sd(df$imic)

df$imic <- df$imic + mn.imic

mean(df$imic)

min(df$imic)

max(df$imic)

sd(df$imic)

cor(df$imic,df$xt1)

# create iMIC2 variable that is correlated with iMIC

( mn.imic2 <- mn.imic2.set*sd(df$xt1) )

( sd.imic2 <- sd.imic2.set*mn.imic )

df$imic2 <- cor.imic.imic2.set*df$imic + sqrt(1-cor.imic.imic2.set^2)*

rnorm(ns,0,sd.imic2)

# Rescale the imic2 variable to the required sd and mean

df$imic2 <- df$imic2 - mean(df$imic2)

df$imic2 <- df$imic2 * sd.imic2 / sd(df$imic2)

df$imic2 <- df$imic2 + mn.imic2

mean(df$imic2)

sd(df$imic2)

cor(df$imic2,df$imic)

cor(df$imic2,df$xt1)

mean(df$imic2 - df$imic)

min(df$imic2 - df$imic) # this one should not be negative!

max(df$imic2 - df$imic)

# Create the "true" HRQOL change score that is correlated with the true T1 score

( sd.xtc.pt <- sd.xtc.set * sd(df$xt1) /2 )

df$xtc.pt1 <- cor.t1.xtc.set*df$xt1.pt1 + sqrt(1-cor.t1.xtc.set^2)*

rnorm(ns,0,sd.xtc.pt)

# Rescale the change score to the required sd and mean

df$xtc.pt1 <- df$xtc.pt1 - mean(df$xtc.pt1)

df$xtc.pt1 <- df$xtc.pt1 * sd.xtc.pt / sd(df$xtc.pt1)

df$xtc.pt1 <- df$xtc.pt1 + (mean(df$imic)/2) + (mn.xtc.set/2) * sd.xtc.pt

df$xtc.pt2 <- df$xtc.pt1 # true change scores are the same

cor(df$xt1.pt1,df$xtc.pt1)

cor(df$xt1.pt2,df$xtc.pt2)

# create true change score

df$xtc <- df$xtc.pt1 + df$xtc.pt2

mean(df$xtc.pt1)

sd(df$xtc.pt1)

mean(df$xtc.pt2)

sd(df$xtc.pt2)

mean(df$xtc)

sd(df$xtc)

cor(df$xt1,df$xtc)

# create "true" HRQOL score at T2

df$xt2.pt1 <- df$xt1.pt1 + df$xtc.pt1

df$xt2.pt2 <- df$xt2.pt1 # true T2 scores are the same

df$xt2 <- df$xt2.pt1 + df$xt2.pt2

mean(df$xt2.pt1)

sd(df$xt2.pt1)

mean(df$xt2.pt2)

sd(df$xt2.pt2)

mean(df$xt2)

sd(df$xt2)

# create GPC values (i.e., proportion improved according to the anchor)

# first, GPC based on the iMIC as benchmark

df$gpc1 <- numeric(ns)

df$gpc1[df$xtc > df$imic] <- 1

mean(df$gpc1)

df$gpc2 <- df$gpc1

df$gpc2[df$xtc > df$imic2] <- 0

mean(df$gpc2) # gpc2=1 is the subgroup "little improved"

table(df$gpc1, df$gpc2)

# create measurement error of the HRQOL measures at T1 and T2;

# add error to the true HRQOL scores at T1 and T2 and

# create (“observed”) HRQOL scores;

# round the T1 and T2 scores

sd.xt.error.pt <- (sqrt(((1-rr.xt.set)/rr.xt.set)*sd(df$xt1)^2))/sqrt(2)

error.t1.pt1 <- rnorm(n=ns, m=0, s=sd.xt.error.pt)

error.t1.pt2 <- rnorm(n=ns, m=0, s=sd.xt.error.pt)

error.t2.pt1 <- rnorm(n=ns, m=0, s=sd.xt.error.pt)

error.t2.pt2 <- rnorm(n=ns, m=0, s=sd.xt.error.pt)

df$xo1.pt1 <- round(df$xt1.pt1 + error.t1.pt1)

df$xo1.pt2 <- round(df$xt1.pt2 + error.t1.pt2)

df$xo2.pt1 <- round(df$xt2.pt1 + error.t2.pt1)

df$xo2.pt2 <- round(df$xt2.pt2 + error.t2.pt2)

df$xo1 <- df$xo1.pt1 + df$xo1.pt2

df$xo2 <- df$xo2.pt1 + df$xo2.pt2

var(df$xt1.pt1)/var(df$xo1.pt1) # reliability of parallel test 1 at T1

var(df$xt2.pt1)/var(df$xo2.pt1) # reliability of parallel test 1 at T2

var(df$xt1.pt2)/var(df$xo1.pt2) # reliability of parallel test 2 at T1

var(df$xt2.pt2)/var(df$xo2.pt2) # reliability of parallel test 2 at T2

var(df$xt1)/var(df$xo1) # reliability of total score T1

var(df$xt2)/var(df$xo2) # reliability of total score T2

# create the HRQOL change score with measurement error

df$xoc.pt1 <- df$xo2.pt1 - df$xo1.pt1

df$xoc.pt2 <- df$xo2.pt2 - df$xo1.pt2

df$xoc <- df$xo2 - df$xo1

# calculate and save the error components of the observed scores

df$xo1.err <- df$xo1 - df$xt1

df$xo2.err <- df$xo2 - df$xt2

df$xoc.err <- df$xoc - df$xtc

mean(df$xo1.err)

mean(df$xo2.err)

mean(df$xoc.err)

df$xo1.pt1.err <- df$xo1.pt1 - df$xt1.pt1

df$xo2.pt1.err <- df$xo2.pt1 - df$xt2.pt1

df$xoc.pt1.err <- df$xoc.pt1 - df$xtc.pt1

mean(df$xo1.pt1.err)

mean(df$xo2.pt1.err)

mean(df$xoc.pt1.err)

df$xo1.pt2.err <- df$xo1.pt2 - df$xt1.pt2

df$xo2.pt2.err <- df$xo2.pt2 - df$xt2.pt2

df$xoc.pt2.err <- df$xoc.pt2 - df$xtc.pt2

mean(df$xo1.pt2.err)

mean(df$xo2.pt2.err)

mean(df$xoc.pt2.err)

cor(df$xo1.pt1, df$xo1.pt2) # test-retest correlation of parallel test T1

# same as reliability of PT score at T1

# calculate proportion improved and log-odds improvement

( q <- mean(df$gpc1) ) # q = proportion improvemed patients (gpc)

( pr <- log(q/(1-q)) ) # log-odds of improvement = log(pre-odds)

## Do logistic regression and calculate MIC(pred) and MIC(adjusted)

( cor.gpc.xoc <- cor(df$gpc1, df$xoc) )

mylogit <- glm(gpc1 ~ xoc, data = df, family = "binomial")

C <- coef(mylogit)[1] # intercept coefficient C

B <- coef(mylogit)[2] # regression coefficient B

( mic.pred <- (pr-C)/B ) # MIC(predicted)

cf <- 0.09 * sd(df$xoc) + 0.103 * sd(df$xoc) * cor.gpc.xoc

( mic.adj <- mic.pred - cf * pr )

## Do ROC analysis and calculate parameters en MIC(ROC)

rocobj <- roc(df$gpc1, df$xoc, quiet = TRUE)

( mic.roc <- coords(rocobj, x="best", input="threshold", ret="threshold",

best.method="youden", transpose = TRUE) )

## Mean change MIC method

( mic.mean <- mean(df[df$gpc2==1,]$xoc) )

## --------------------------------

## Results Table 3, column 6

mean(df$gpc1)

mean(df$gpc2)

mean(df$imic)

mean(df$imic2)

cor(df$xt1,df$xtc)

cor(df$xt1,df$imic)

mean(df$xo1)

mean(df$xo1.err)

mean(df$xo2)

mean(df$xo2.err)

mean(df$xoc)

mean(df$xoc.err)

mic.mean

mic.roc

mic.pred

mic.adj

#########################################################################

## Median split dataset PT1 on baseline score of PT2

( med.coff <- median(df$xo1.pt2) )

df1 <- df[df$xo1.pt2 < med.coff,] # low baseline subgroup

df2 <- df[df$xo1.pt2 >= med.coff,] # high baseline subgroup

summary(df1$xo1.pt1)

summary(df2$xo1.pt2)

ggplot(df1, aes(x=xo1.pt1)) +

geom_histogram(binwidth=1, colour="black", fill="white") +

dev.new(width=5, height=4)

ggplot(df2, aes(x=xo1.pt1)) +

geom_histogram(binwidth=1, colour="black", fill="white") +

dev.new(width=5, height=4)

###### MICs low baseline group

( q1 <- mean(df1$gpc1) ) # q = proportion improvemed patients (gpc)

( pr1 <- log(q1/(1-q1)) ) # log-odds of improvement = log(pre-odds)

( cor.gpc.xoc.1 <- cor(df1$gpc1, df1$xoc.pt1) )

## Calculate MIC(pred) and MIC(adjusted) for df1

mylogit <- glm(gpc1 ~ xoc.pt1, data = df1, family = "binomial")

C <- coef(mylogit)[1] # intercept coefficient C

B <- coef(mylogit)[2] # regression coefficient B

( mic.pred.1 <- (pr1-C)/B ) # MIC(predicted)

cf <- 0.09 * sd(df1$xoc.pt1) + 0.103 * sd(df1$xoc.pt1) * cor.gpc.xoc.1

( mic.adj.1 <- mic.pred.1 - cf * pr1 )

## Calculate MIC(ROC) for df1

rocobj <- roc(df1$gpc1, df1$xoc.pt1, quiet = TRUE)

( mic.roc.1 <- coords(rocobj, x="best", input="threshold", ret="threshold",

best.method="youden", transpose = TRUE) )

## Mean change MIC method

( mic.mean.1 <- mean(df1[df1$gpc2==1,]$xoc.pt1) ) # mean change MIC group 1

###### MICs high baseline group

## Calculate MIC(pred) and MIC(adjusted) for df2

( q2 <- mean(df2$gpc1) ) # q = proportion improvemed patients (gpc)

( pr2 <- log(q2/(1-q2)) ) # log-odds of improvement = log(pre-odds)

( cor.gpc.xoc.2 <- cor(df2$gpc1, df2$xoc.pt2) )

mylogit <- glm(gpc1 ~ xoc.pt1, data = df2, family = "binomial")

C <- coef(mylogit)[1] # intercept coefficient C

B <- coef(mylogit)[2] # regression coefficient B

( mic.pred.2 <- (pr2-C)/B ) # MIC(predicted)

cf <- 0.09 * sd(df2$xoc.pt1) + 0.103 * sd(df2$xoc.pt1) * cor.gpc.xoc.2

( mic.adj.2 <- mic.pred.2 - cf * pr2 )

## Calculate MIC(ROC) for df2

rocobj <- roc(df2$gpc1, df2$xoc.pt1, quiet = TRUE)

( mic.roc.2 <- coords(rocobj, x="best", input="threshold", ret="threshold",

best.method="youden", transpose = TRUE) )

## Mean change MIC method

( mic.mean.2 <- mean(df2[df2$gpc2==1,]$xoc.pt1) ) # mean change MIC group 2

# Table 3, column 7

mean(df1$gpc1)

mean(df1$gpc2)

mean(df1$imic)

mean(df1$imic2)

cor(df1$xt1.pt1,df1$xtc.pt1)

cor(df1$xt1.pt1,df1$imic)

mean(df1$xo1.pt1)

mean(df1$xo1.pt1.err)

mean(df1$xo2.pt1)

mean(df1$xo2.pt1.err)

mean(df1$xoc.pt1)

mean(df1$xoc.pt1.err)

mic.mean.1

mic.roc.1

mic.pred.1

mic.adj.1

# Table 3, column 8

mean(df2$gpc1)

mean(df2$gpc2)

mean(df2$imic)

mean(df2$imic2)

cor(df2$xt1.pt1,df2$xtc.pt1)

cor(df2$xt1.pt1,df2$imic)

mean(df2$xo1.pt1)

mean(df2$xo1.pt1.err)

mean(df2$xo2.pt1)

mean(df2$xo2.pt1.err)

mean(df2$xoc.pt1)

mean(df2$xoc.pt1.err)

mic.mean.2

mic.roc.2

mic.pred.2

mic.adj.2

################################################################

## Median split dataset PT2 on baseline score of PT1

( med.coff <- median(df$xo1.pt1) )

df1 <- df[df$xo1.pt1 < med.coff,] # low baseline subgroup

df2 <- df[df$xo1.pt1 >= med.coff,] # high baseline subgroup

summary(df1$xo1.pt2)

summary(df2$xo1.pt2)

ggplot(df1, aes(x=xo1.pt2)) +

geom_histogram(binwidth=1, colour="black", fill="white") +

dev.new(width=5, height=4)

ggplot(df2, aes(x=xo1.pt2)) +

geom_histogram(binwidth=1, colour="black", fill="white") +

dev.new(width=5, height=4)

###### MICs low baseline group

## Calculate MIC(pred) and MIC(adjusted) for df1

( q1 <- mean(df1$gpc1) ) # q = proportion improvemed patients (gpc)

( pr1 <- log(q1/(1-q1)) ) # log-odds of improvement = log(pre-odds)

( cor.gpc.xoc.1 <- cor(df1$gpc1, df1$xoc.pt2) )

mylogit <- glm(gpc1 ~ xoc.pt2, data = df1, family = "binomial")

C <- coef(mylogit)[1] # intercept coefficient C

B <- coef(mylogit)[2] # regression coefficient B

( mic.pred.1 <- (pr1-C)/B ) # MIC(predicted)

cf <- 0.09 * sd(df1$xoc.pt2) + 0.103 * sd(df1$xoc.pt2) * cor.gpc.xoc.1

( mic.adj.1 <- mic.pred.1 - cf * pr1 )

## Calculate MIC(ROC) for df1

rocobj <- roc(df1$gpc1, df1$xoc.pt2, quiet = TRUE)

( mic.roc.1 <- coords(rocobj, x="best", input="threshold", ret="threshold",

best.method="youden", transpose = TRUE) )

## Mean change MIC method

( mic.mean.1 <- mean(df1[df1$gpc2==1,]$xoc.pt2) ) # mean change MIC group 1

###### MICs high baseline group

## Calculate MIC(pred) and MIC(adjusted) for df2

( q2 <- mean(df2$gpc1) ) # q = proportion improvemed patients (gpc)

( pr2 <- log(q2/(1-q2)) ) # log-odds of improvement = log(pre-odds)

( cor.gpc.xoc.2 <- cor(df2$gpc1, df2$xoc.pt1) )

mylogit <- glm(gpc1 ~ xoc.pt2, data = df2, family = "binomial")

C <- coef(mylogit)[1] # intercept coefficient C

B <- coef(mylogit)[2] # regression coefficient B

( mic.pred.2 <- (pr2-C)/B ) # MIC(predicted)

cf <- 0.09 * sd(df2$xoc.pt2) + 0.103 * sd(df2$xoc.pt2) * cor.gpc.xoc.2

( mic.adj.2 <- mic.pred.2 - cf * pr2 )

## Calculate MIC(ROC) for df2

rocobj <- roc(df2$gpc1, df2$xoc.pt2, quiet = TRUE)

( mic.roc.2 <- coords(rocobj, x="best", input="threshold", ret="threshold",

best.method="youden", transpose = TRUE) )

## Mean change MIC method

mean(df2[df2$gpc2==1,]$xoc.pt2) # mean change MIC group 2

# Table 3, column 9

mean(df1$gpc1)

mean(df1$gpc2)

mean(df1$imic)

mean(df1$imic2)

cor(df1$xt1.pt2,df1$xtc.pt2)

cor(df1$xt1.pt2,df1$imic)

mean(df1$xo1.pt2)

mean(df1$xo1.pt2.err)

mean(df1$xo2.pt2)

mean(df1$xo2.pt2.err)

mean(df1$xoc.pt2)

mean(df1$xoc.pt2.err)

mic.mean.1

mic.roc.1

mic.pred.1

mic.adj.1

# Table 3, column 10

mean(df2$gpc1)

mean(df2$gpc2)

mean(df2$imic)

mean(df2$imic2)

cor(df2$xt1.pt2,df2$xtc.pt2)

cor(df2$xt1.pt2,df2$imic)

mean(df2$xo1.pt2)

mean(df2$xo1.pt2.err)

mean(df2$xo2.pt2)

mean(df2$xo2.pt2.err)

mean(df2$xoc.pt2)

mean(df2$xoc.pt2.err)

mic.mean.2

mic.roc.2

mic.pred.2

mic.adj.2

#########################################################################

Section 3

**R-code for the item-split method to assess MIC baseline dependency, with bootstrapping**

*Manual*

The following R-code can be executed in the statistical program R. You don’t have to be familiar with R to be able to use this code.

R is freeware and can be downloaded and installed from <https://www.r-project.org/>. There are R-versions for Windows, Mac OS X and Linux.

Once you have installed R on your computer, open it and choose “File” > “New script …” from the menu. A blank window appears. Copy the code below and paste the text in the New script window in R.

Next, you need to install the R-packages pROC (to calculate ROC-based MICs) and mirt (if you want to simulate data). Choose “Packages” from the menu bar and choose “Install packages …”. Choose a suitable country from the list of CRAN mirrors, and then choose the package one by one from the list and click OK.

Part 1 allows you to simulate an item response dataset with transition ratings, fit for estimating MICs.

Part 2 allows you to analyse your own dataset.

Beware that you must provide your data in the correct format, which is:

- Time 1 items first,

- then Time 2 items (in the same order),

- then the dichotomous transition item (0 for not improved, i.e., unchanged or worse, and 1 for improved, i.e., little improved, much improved, and recovered),

- then the polytomous transition item (0 for not improved, i.e., unchanged or worse, 1 for little improved, and 2 for much improved and recovered).

######################################################################

#### ####

#### BASELINE DEPENDENCY METHOD 3 (ITEM-SPLIT METHOD) ####

#### ####

######################################################################

############################### PART 1 #############################

# Part 1 contains the code to simulate a dataset with 25 items.

# If you want to analyze your own dataset, go to Part 2.

# Remove all objects

rm(list=ls(all=TRUE))

# Acquire package

library(mirt) # for simulating data

library(pROC) # for ROC analysis

## Simulate a set of IRT item parameters

set.seed(12345)

nitems <- 25

b1 <- rep(-3,nitems)

b2 <- rep(-1,nitems)

b3 <- rep(1,nitems)

b4 <- rep(3,nitems)

b1 <- b1 + runif(nitems,-0.75,0.75)

b2 <- b2 + runif(nitems,-0.75,0.75)

b3 <- b3 + runif(nitems,-0.75,0.75)

b4 <- b4 + runif(nitems,-0.75,0.75)

a1 <- runif(nitems,0.5,1)

cf.simb <- data.frame(a1,b1,b2,b3,b4)

# Transform b-parameters to d-parameters (mirt works with d-parameters)

# difficulty (b) = easiness (d) / -a

cf.sim <- cf.simb

colnames(cf.sim) <- c("a1","d1","d2","d3","d4")

cf.sim$d1 <- -cf.simb$b1*cf.sim$a1

cf.sim$d2 <- -cf.simb$b2*cf.sim$a1

cf.sim$d3 <- -cf.simb$b3*cf.sim$a1

cf.sim$d4 <- -cf.simb$b4*cf.sim$a1

round(cf.sim, 3)

round(colMeans(cf.sim), 3)

a1 <- as.matrix(cf.sim[ , 1])

d1 <- as.matrix(cf.sim[ , -1])

# Create thetas T1 and T2

N <- 300

theta1 <- as.matrix(rnorm(N, 0, 1))

thetach <- as.matrix(rnorm(N, 0.75, 1))

theta2 <- theta1 + thetach

# Simulate datasets T1 and T2 using 'mirt'

dat1 <- simdata(a1, d1, itemtype="graded", Theta=theta1)

dat1 <- as.data.frame(dat1)

dat2 <- simdata(a1, d1, itemtype="graded", Theta=theta2)

dat2 <- as.data.frame(dat2)

# Create transition ratings, both ploytomous and dichotomous

tr.pol <- numeric(N)

tr.pol[thetach>0.75] <- 1

tr.pol[thetach>1.25] <- 2

length(tr.pol[tr.pol==1])/N # proportion "a little better"

tr.dich <- numeric(N)

tr.dich[thetach>0.75] <- 1

mean(tr.dich) # proportion improved

# Put the items in 1 file

datw <- data.frame(dat1, dat2, tr.dich, tr.pol)

# data.frame(names(datw))

############################### PART 2 #############################

### Read your own data

# The following code opens a windows dialogue box in which you can browse

# to your data file.

# Note that your data (text) file should contain the items of T1 and T2

# (items in the same order) and in the last 2 columns the transition ratings.

# First, the dichotomous TR distinguishing (all improved and all not-improved

# i.e., unchanged and deteriorated.

# Second, a TR with 3 categories: '0' for unchanged or deteriorated, '1' for

# a little better, and '2' for much better or recovered.

# You can use any variable names you like.

# Don't include scale scores or change scores in your file.

datw <- read.table(file.choose(), header=T) # The file is named "datw"

# Remove cases with missing values, if necessary

datw <- datw[complete.cases(datw),]

############################### PART 3 #############################

nitems <- 25 # number of items -- ADJUST THIS NUMBER IF NECESSARY

noptions <- 5 # number of response options per item -- ADJUST IF NEC.

ns <- 5 # number of times to split the items randomly

mic.pred.low <- as.numeric(rep(NA, ns)) # MIC predicted

mic.pred.hig <- as.numeric(rep(NA, ns))

mic.adj.low <- as.numeric(rep(NA, ns)) # MIC adjusted

mic.adj.hig <- as.numeric(rep(NA, ns))

mic.roc.low <- as.numeric(rep(NA, ns)) # MIC ROC

mic.roc.hig <- as.numeric(rep(NA, ns))

mic.mean.low <- as.numeric(rep(NA, ns)) # MIC mean change

mic.mean.hig <- as.numeric(rep(NA, ns))

########### Start FOR-loop ##########

for (i in 1:ns) {

( nitems.pt1 <- round(nitems/2) ) # number of items parallel test 1

( nitems.pt2 <- nitems - nitems.pt1 ) # number of items parallel test 2

( items.pt1 <- sample(1:nitems,nitems.pt1) ) # random selection of items in set 1

items.pt2 <- 1:nitems

( items.pt2 <- items.pt2[ - items.pt1] ) # selection of items in set 2

# Split datw along 2 sets of items / 2 parallel tests

datw.pt1 <- datw[,c(items.pt1, items.pt1+nitems, 2*nitems+1, 2*nitems+2)]

datw.pt2 <- datw[,c(items.pt2, items.pt2+nitems, 2*nitems+1, 2*nitems+2)]

################ ANALYSE FIRST ITEM SET / PARALLEL TEST

##### Split datw.pt1 into low and high baseline

### Use datw.pt2 to select baseline subgroups of datw.pt1

xo1.pt2 <- rowSums(datw.pt2[,1:nitems.pt2]) # Sumscore at T2

( med.xo1.pt2 <- median(xo1.pt2) ) # median sumscore T2

datw.pt1.L <- datw.pt1[xo1.pt2 < med.xo1.pt2,] # Low baseline subgroup

datw.pt1.H <- datw.pt1[xo1.pt2 >= med.xo1.pt2,] # High baseline subgroup

############ LOW BASELINE, parallel test 1

## Do logistic regression and calculate MIC-predictive

( pi <- mean(datw.pt1.L[,2*nitems.pt1+1]) )

( logoddspi <- log(pi/(1-pi)) ) # log-odds of improvement

trt <- datw.pt1.L[,2*nitems.pt1+1]

xo1 <- rowSums(datw.pt1.L[,1:nitems.pt1])

xo2 <- rowSums(datw.pt1.L[,(nitems.pt1+1):(nitems.pt1*2)])

xoc <- xo2-xo1

( cor.trch <- cor(xoc,trt) )

fit <- glm(trt ~ xoc, family = "binomial")

( C <- coef(fit)[1] ) # intercept coefficient C

( B <- coef(fit)[2] ) # regression coefficient B

( mic.pred.pt1.L <- (logoddspi - C)/B ) # MIC(predicted)

cf <- 0.09 * sd(xoc) + 0.103 * sd(xoc) * cor.trch

( mic.adj.pt1.L <- mic.pred.pt1.L - cf * logoddspi )

## ROC-based MIC

rocobj <- roc(trt, xoc, quiet = TRUE)

cuty <- coords(rocobj, x="best", input="threshold", ret="threshold",

best.method="youden", transpose = TRUE)

( mic.roc.pt1.L <- cuty[sample(1:length(cuty),1)] )

## MIC mean change

trt2 <- datw.pt1.L[,2*nitems.pt1+2]

( mic.mean.pt1.L <- mean(xoc[trt2==1]) )

length(trt2[trt2==1])/nrow(datw.pt1.L) # proportion "a little better"

############ HIGH Baseline, parallel test 1

## Do logistic regression and calculate MIC-predictive

( pi <- mean(datw.pt1.H[,2*nitems.pt1+1]) )

( logoddspi <- log(pi/(1-pi)) ) # log-odds of improvement

trt <- datw.pt1.H[,2*nitems.pt1+1]

xo1 <- rowSums(datw.pt1.H[,1:nitems.pt1])

xo2 <- rowSums(datw.pt1.H[,(nitems.pt1+1):(nitems.pt1*2)])

xoc <- xo2-xo1

( cor.trch <- cor(xoc,trt) )

fit <- glm(trt ~ xoc, family = "binomial")

( C <- coef(fit)[1] ) # intercept coefficient C

( B <- coef(fit)[2] ) # regression coefficient B

( mic.pred.pt1.H <- (logoddspi - C)/B ) # MIC(predicted)

cf <- 0.09 * sd(xoc) + 0.103 * sd(xoc) * cor.trch

( mic.adj.pt1.H <- mic.pred.pt1.H - cf * logoddspi )

## ROC-based MIC

rocobj <- roc(trt, xoc, quiet = TRUE)

cuty <- coords(rocobj, x="best", input="threshold", ret="threshold",

best.method="youden", transpose = TRUE)

( mic.roc.pt1.H <- cuty[sample(1:length(cuty),1)] )

## MIC mean change

trt2 <- datw.pt1.H[,2*nitems.pt1+2]

( mic.mean.pt1.H <- mean(xoc[trt2==1]) )

length(trt2[trt2==1])/nrow(datw.pt1.H) # proportion "a little better"

################ ANALYSE SECOND ITEM SET / PARALLEL TEST

### Use datw.pt1 to select severity subgroups of datw.pt2

xo1.pt1 <- rowSums(datw.pt1[,1:nitems.pt1])

( med.xo1.pt1 <- median(xo1.pt1) ) # median sumscore item set 1

# Split datw.pt2 into low and high baseline

datw.pt2.L <- datw.pt2[xo1.pt1 < med.xo1.pt1,]

datw.pt2.H <- datw.pt2[xo1.pt1 >= med.xo1.pt1,]

### LOW Baseline, parallel test 2

## Do logistic regression and calculate MIC-predictive

( pi <- mean(datw.pt2.L[,2*nitems.pt2+1]) )

( logoddspi <- log(pi/(1-pi)) ) # log-odds of improvement

trt <- datw.pt2.L[,2*nitems.pt2+1]

xo1 <- rowSums(datw.pt2.L[,1:nitems.pt2])

xo2 <- rowSums(datw.pt2.L[,(nitems.pt2+1):(nitems.pt2*2)])

xoc <- xo2-xo1

( cor.trch <- cor(xoc,trt) )

fit <- glm(trt ~ xoc, family = "binomial")

( C <- coef(fit)[1] ) # intercept coefficient C

( B <- coef(fit)[2] ) # regression coefficient B

( mic.pred.pt2.L <- (logoddspi - C)/B ) # MIC(predicted)

cf <- 0.09 * sd(xoc) + 0.103 * sd(xoc) * cor.trch

( mic.adj.pt2.L <- mic.pred.pt2.L - cf * logoddspi )

## ROC-based MIC

rocobj <- roc(trt, xoc, quiet = TRUE)

cuty <- coords(rocobj, x="best", input="threshold", ret="threshold",

best.method="youden", transpose = TRUE)

( mic.roc.pt2.L <- cuty[sample(1:length(cuty),1)] )

## MIC mean change

trt2 <- datw.pt2.L[,2*nitems.pt2+2]

( mic.mean.pt2.L <- mean(xoc[trt2==1]) )

length(trt2[trt2==1])/nrow(datw.pt2.L) # proportion "a little better"

### HIGH Baseline, parallel test 2

## Do logistic regression and calculate MIC-predictive

( pi <- mean(datw.pt2.H[,2*nitems.pt2+1]) )

( logoddspi <- log(pi/(1-pi)) ) # log-odds of improvement

trt <- datw.pt2.H[,2*nitems.pt2+1]

xo1 <- rowSums(datw.pt2.H[,1:nitems.pt2])

xo2 <- rowSums(datw.pt2.H[,(nitems.pt2+1):(nitems.pt2*2)])

xoc <- xo2-xo1

( cor.trch <- cor(xoc,trt) )

fit <- glm(trt ~ xoc, family = "binomial")

( C <- coef(fit)[1] ) # intercept coefficient C

( B <- coef(fit)[2] ) # regression coefficient B

( mic.pred.pt2.H <- (logoddspi - C)/B ) # MIC(predicted)

cf <- 0.09 * sd(xoc) + 0.103 * sd(xoc) * cor.trch

( mic.adj.pt2.H <- mic.pred.pt2.H - cf * logoddspi )

## ROC-based MIC

rocobj <- roc(trt, xoc, quiet = TRUE)

cuty <- coords(rocobj, x="best", input="threshold", ret="threshold",

best.method="youden", transpose = TRUE)

( mic.roc.pt2.H <- cuty[sample(1:length(cuty),1)] )

## MIC mean change

trt2 <- datw.pt2.H[,2*nitems.pt2+2]

( mic.mean.pt2.H <- mean(xoc[trt2==1]) )

length(trt2[trt2==1])/nrow(datw.pt2.H) # proportion "a little better"

####### Combine results of both splits

( mic.pred.low[i] <- mic.pred.pt1.L + mic.pred.pt2.L )

( mic.pred.hig[i] <- mic.pred.pt1.H + mic.pred.pt2.H )

( mic.adj.low[i] <- mic.adj.pt1.L + mic.adj.pt2.L )

( mic.adj.hig[i] <- mic.adj.pt1.H + mic.adj.pt2.H )

( mic.roc.low[i] <- mic.roc.pt1.L + mic.roc.pt2.L )

( mic.roc.hig[i] <- mic.roc.pt1.H + mic.roc.pt2.H )

( mic.mean.low[i] <- mic.mean.pt1.L + mic.mean.pt2.L )

( mic.mean.hig[i] <- mic.mean.pt1.H + mic.mean.pt2.H )

}

####### End FOR-loop ###########

## Results of the one-time estimates in the original dataset

round( mean(mic.pred.low),1) # mean MIC predicted for low severity groups

round( mean(mic.pred.hig),1) # mean MIC predicted for high severity groups

round( mean(mic.adj.low),1) # MIC adjusted

round( mean(mic.adj.hig),1)

round( mean(mic.roc.low),1) # MIC ROC

round( mean(mic.roc.hig),1)

round( mean(mic.mean.low),1) # MIC mean

round( mean(mic.mean.hig),1)

#######################################################################

############# BOOTSTRAPPEN #########################################

org <- datw

set.seed (12345)

nb <- 1000 # set number of bootstrap samples

boot.mic.pred.low <- as.numeric(rep(NA, nb))

boot.mic.pred.hig <- as.numeric(rep(NA, nb))

boot.mic.pred.dif <- as.numeric(rep(NA, nb))

boot.mic.adj.low <- as.numeric(rep(NA, nb))

boot.mic.adj.hig <- as.numeric(rep(NA, nb))

boot.mic.adj.dif <- as.numeric(rep(NA, nb))

boot.mic.roc.low <- as.numeric(rep(NA, nb))

boot.mic.roc.hig <- as.numeric(rep(NA, nb))

boot.mic.roc.dif <- as.numeric(rep(NA, nb))

boot.mic.mean.low <- as.numeric(rep(NA, nb))

boot.mic.mean.hig <- as.numeric(rep(NA, nb))

boot.mic.mean.dif <- as.numeric(rep(NA, nb))

#### START BOOTSTRAP LOOP ####

for(k in 1:nb) {

datw <- org[sample(1:nrow(org), nrow(org), replace=TRUE),]

trt2 <- datw[,2*nitems]

# prevent continuation with 0% or 100% improved cases

for(j in 1:10) {

if(mean(datw[,2*nitems+1])==0 |

mean(datw[,2*nitems+1])==1 |

length(trt2[trt2==1])==0 ) {

datw <- org[sample(1:nrow(org), nrow(org), replace=TRUE),]

trt2 <- datw[,2*nitems]

}

}

print(k) # While running the analysis, if you click on the R Console

# the number of the sample that is analyzed is displayed

########### Start FOR-loop ##########

for (i in 1:ns) {

( nitems.pt1 <- round(nitems/2) ) # number of items parallel test 1

( nitems.pt2 <- nitems - nitems.pt1 ) # number of items parallel test 2

( items.pt1 <- sample(1:nitems,nitems.pt1) ) # random selection of items in set 1

items.pt2 <- 1:nitems

( items.pt2 <- items.pt2[ - items.pt1] ) # selection of items in set 2

# Split datw along 2 sets of items / 2 parallel tests

datw.pt1 <- datw[,c(items.pt1, items.pt1+nitems, 2*nitems+1, 2*nitems+2)]

datw.pt2 <- datw[,c(items.pt2, items.pt2+nitems, 2*nitems+1, 2*nitems+2)]

################ ANALYSE FIRST ITEM SET / PARALLEL TEST

##### Split datw.pt1 into low and high baseline

### Use datw.pt2 to select baseline subgroups of datw.pt1

xo1.pt2 <- rowSums(datw.pt2[,1:nitems.pt2]) # Sumscore at T2

( med.xo1.pt2 <- median(xo1.pt2) ) # median sumscore T2

datw.pt1.L <- datw.pt1[xo1.pt2 < med.xo1.pt2,] # Low baseline subgroup

datw.pt1.H <- datw.pt1[xo1.pt2 >= med.xo1.pt2,] # High baseline subgroup

############ LOW BASELINE, parallel test 1

## Do logistic regression and calculate MIC-predictive

( pi <- mean(datw.pt1.L[,2*nitems.pt1+1]) )

( logoddspi <- log(pi/(1-pi)) ) # log-odds of improvement

trt <- datw.pt1.L[,2*nitems.pt1+1]

xo1 <- rowSums(datw.pt1.L[,1:nitems.pt1])

xo2 <- rowSums(datw.pt1.L[,(nitems.pt1+1):(nitems.pt1*2)])

xoc <- xo2-xo1

( cor.trch <- cor(xoc,trt) )

fit <- glm(trt ~ xoc, family = "binomial")

( C <- coef(fit)[1] ) # intercept coefficient C

( B <- coef(fit)[2] ) # regression coefficient B

( mic.pred.pt1.L <- (logoddspi - C)/B ) # MIC(predicted)

cf <- 0.09 * sd(xoc) + 0.103 * sd(xoc) * cor.trch

( mic.adj.pt1.L <- mic.pred.pt1.L - cf * logoddspi )

## ROC-based MIC

rocobj <- roc(trt, xoc, quiet = TRUE)

cuty <- coords(rocobj, x="best", input="threshold", ret="threshold",

best.method="youden", transpose = TRUE)

( mic.roc.pt1.L <- cuty[sample(1:length(cuty),1)] )

## MIC mean change

trt2 <- datw.pt1.L[,2*nitems.pt1+2]

( mic.mean.pt1.L <- mean(xoc[trt2==1]) )

length(trt2[trt2==1])/nrow(datw.pt1.L) # proportion "a little better"

############ HIGH Baseline, parallel test 1

## Do logistic regression and calculate MIC-predictive

( pi <- mean(datw.pt1.H[,2*nitems.pt1+1]) )

( logoddspi <- log(pi/(1-pi)) ) # log-odds of improvement

trt <- datw.pt1.H[,2*nitems.pt1+1]

xo1 <- rowSums(datw.pt1.H[,1:nitems.pt1])

xo2 <- rowSums(datw.pt1.H[,(nitems.pt1+1):(nitems.pt1*2)])

xoc <- xo2-xo1

( cor.trch <- cor(xoc,trt) )

fit <- glm(trt ~ xoc, family = "binomial")

( C <- coef(fit)[1] ) # intercept coefficient C

( B <- coef(fit)[2] ) # regression coefficient B

( mic.pred.pt1.H <- (logoddspi - C)/B ) # MIC(predicted)

cf <- 0.09 * sd(xoc) + 0.103 * sd(xoc) * cor.trch

( mic.adj.pt1.H <- mic.pred.pt1.H - cf * logoddspi )

## ROC-based MIC

rocobj <- roc(trt, xoc, quiet = TRUE)

cuty <- coords(rocobj, x="best", input="threshold", ret="threshold",

best.method="youden", transpose = TRUE)

( mic.roc.pt1.H <- cuty[sample(1:length(cuty),1)] )

## MIC mean change

trt2 <- datw.pt1.H[,2*nitems.pt1+2]

( mic.mean.pt1.H <- mean(xoc[trt2==1]) )

length(trt2[trt2==1])/nrow(datw.pt1.H) # proportion "a little better"

################ ANALYSE SECOND ITEM SET / PARALLEL TEST

### Use datw.pt1 to select severity subgroups of datw.pt2

xo1.pt1 <- rowSums(datw.pt1[,1:nitems.pt1])

( med.xo1.pt1 <- median(xo1.pt1) ) # median sumscore item set 1

# Split datw.pt2 into low and high baseline

datw.pt2.L <- datw.pt2[xo1.pt1 < med.xo1.pt1,]

datw.pt2.H <- datw.pt2[xo1.pt1 >= med.xo1.pt1,]

### LOW Baseline, parallel test 2

## Do logistic regression and calculate MIC-predictive

( pi <- mean(datw.pt2.L[,2*nitems.pt2+1]) )

( logoddspi <- log(pi/(1-pi)) ) # log-odds of improvement

trt <- datw.pt2.L[,2*nitems.pt2+1]

xo1 <- rowSums(datw.pt2.L[,1:nitems.pt2])

xo2 <- rowSums(datw.pt2.L[,(nitems.pt2+1):(nitems.pt2*2)])

xoc <- xo2-xo1

( cor.trch <- cor(xoc,trt) )

fit <- glm(trt ~ xoc, family = "binomial")

( C <- coef(fit)[1] ) # intercept coefficient C

( B <- coef(fit)[2] ) # regression coefficient B

( mic.pred.pt2.L <- (logoddspi - C)/B ) # MIC(predicted)

cf <- 0.09 * sd(xoc) + 0.103 * sd(xoc) * cor.trch

( mic.adj.pt2.L <- mic.pred.pt2.L - cf * logoddspi )

## ROC-based MIC

rocobj <- roc(trt, xoc, quiet = TRUE)

cuty <- coords(rocobj, x="best", input="threshold", ret="threshold",

best.method="youden", transpose = TRUE)

( mic.roc.pt2.L <- cuty[sample(1:length(cuty),1)] )

## MIC mean change

trt2 <- datw.pt2.L[,2*nitems.pt2+2]

( mic.mean.pt2.L <- mean(xoc[trt2==1]) )

length(trt2[trt2==1])/nrow(datw.pt2.L) # proportion "a little better"

### HIGH Baseline, parallel test 2

## Do logistic regression and calculate MIC-predictive

( pi <- mean(datw.pt2.H[,2*nitems.pt2+1]) )

( logoddspi <- log(pi/(1-pi)) ) # log-odds of improvement

trt <- datw.pt2.H[,2*nitems.pt2+1]

xo1 <- rowSums(datw.pt2.H[,1:nitems.pt2])

xo2 <- rowSums(datw.pt2.H[,(nitems.pt2+1):(nitems.pt2*2)])

xoc <- xo2-xo1

( cor.trch <- cor(xoc,trt) )

fit <- glm(trt ~ xoc, family = "binomial")

( C <- coef(fit)[1] ) # intercept coefficient C

( B <- coef(fit)[2] ) # regression coefficient B

( mic.pred.pt2.H <- (logoddspi - C)/B ) # MIC(predicted)

cf <- 0.09 * sd(xoc) + 0.103 * sd(xoc) * cor.trch

( mic.adj.pt2.H <- mic.pred.pt2.H - cf * logoddspi )

## ROC-based MIC

rocobj <- roc(trt, xoc, quiet = TRUE)

cuty <- coords(rocobj, x="best", input="threshold", ret="threshold",

best.method="youden", transpose = TRUE)

( mic.roc.pt2.H <- cuty[sample(1:length(cuty),1)] )

## MIC mean change

trt2 <- datw.pt2.H[,2*nitems.pt2+2]

( mic.mean.pt2.H <- mean(xoc[trt2==1]) )

length(trt2[trt2==1])/nrow(datw.pt2.H) # proportion "a little better"

####### Combine results of both splits

( mic.pred.low[i] <- mic.pred.pt1.L + mic.pred.pt2.L )

( mic.pred.hig[i] <- mic.pred.pt1.H + mic.pred.pt2.H )

( mic.adj.low[i] <- mic.adj.pt1.L + mic.adj.pt2.L )

( mic.adj.hig[i] <- mic.adj.pt1.H + mic.adj.pt2.H )

( mic.roc.low[i] <- mic.roc.pt1.L + mic.roc.pt2.L )

( mic.roc.hig[i] <- mic.roc.pt1.H + mic.roc.pt2.H )

( mic.mean.low[i] <- mic.mean.pt1.L + mic.mean.pt2.L )

( mic.mean.hig[i] <- mic.mean.pt1.H + mic.mean.pt2.H )

}

####### End FOR-loop ###########

boot.mic.pred.low[k] <- mean(mic.pred.low) # MIC predicted

boot.mic.pred.hig[k] <- mean(mic.pred.hig)

boot.mic.pred.dif[k] <- boot.mic.pred.low[k]-boot.mic.pred.hig[k]

boot.mic.adj.low[k] <- mean(mic.adj.low) # MIC adjusted

boot.mic.adj.hig[k] <- mean(mic.adj.hig)

boot.mic.adj.dif[k] <- boot.mic.adj.low[k]-boot.mic.adj.hig[k]

boot.mic.roc.low[k] <- mean(mic.roc.low) # MIC ROC

boot.mic.roc.hig[k] <- mean(mic.roc.hig)

boot.mic.roc.dif[k] <- boot.mic.roc.low[k]-boot.mic.roc.hig[k]

boot.mic.mean.low[k] <- mean(mic.mean.low) # MIC mean

boot.mic.mean.hig[k] <- mean(mic.mean.hig)

boot.mic.mean.dif[k] <- boot.mic.mean.low[k]-boot.mic.mean.hig[k]

}

####### END BOOTSTRAP LOOP #######

## BOOTSTRAP RESULTS: MEANS

# MIC predicted

round( mean(boot.mic.pred.low),1) # Low baseline

round( mean(boot.mic.pred.hig),1) # High baseline

round( mean(boot.mic.pred.dif),1) # L-H difference

# MIC adjusted

round( mean(boot.mic.adj.low),1)

round( mean(boot.mic.adj.hig),1)

round( mean(boot.mic.adj.dif),1)

# MIC ROC

round( mean(boot.mic.roc.low),1)

round( mean(boot.mic.roc.hig),1)

round( mean(boot.mic.roc.dif),1)

# MIC mean

round( mean(boot.mic.mean.low),1)

round( mean(boot.mic.mean.hig),1)

round( mean(boot.mic.mean.dif),1)

## BOOTSTRAP RESULTS: 95% CONFIDENCE INTERVALS

# MIC predicted

round( quantile(boot.mic.pred.low, c(0.025, 0.975)),1) # Low baseline

round( quantile(boot.mic.pred.hig, c(0.025, 0.975)),1) # High baseline

round( quantile(boot.mic.pred.dif, c(0.025, 0.975)),1) # L-H difference

# MIC adjusted

round( quantile(boot.mic.adj.low, c(0.025, 0.975)),1)

round( quantile(boot.mic.adj.hig, c(0.025, 0.975)),1)

round( quantile(boot.mic.adj.dif, c(0.025, 0.975)),1)

# MIC ROC

round( quantile(boot.mic.roc.low, c(0.025, 0.975)),1)

round( quantile(boot.mic.roc.hig, c(0.025, 0.975)),1)

round( quantile(boot.mic.roc.dif, c(0.025, 0.975)),1)

# MIC mean

round( quantile(boot.mic.mean.low, c(0.025, 0.975)),1)

round( quantile(boot.mic.mean.hig, c(0.025, 0.975)),1)

round( quantile(boot.mic.mean.dif, c(0.025, 0.975)),1)

#######################################################################
